# Combinatorial TGF-β attenuation with paclitaxel inhibits the epithelial-to-mesenchymal transition and breast cancer stem-like cells

**DOI:** 10.18632/oncotarget.6063

**Published:** 2015-10-09

**Authors:** So-Yeon Park, Min-Jin Kim, Sang-A Park, Jung-Shin Kim, Kyung-Nan Min, Dae-Kee Kim, Woosung Lim, Jeong-Seok Nam, Yhun Yhong Sheen

**Affiliations:** ^1^ College of Pharmacy, Ewha Womans University, Seoul, South Korea; ^2^ Pharmaceutical Examination Division, Korean Intellectual Property Office, Daejeon, South Korea; ^3^ Department of Surgery, School of Medicine, Ewha Womans University, Mokdong Hospital, Seoul, South Korea; ^4^ Laboratory of Tumor Suppressor, Lee Gil Ya Cancer and Diabetes Institute, Gachon University, Incheon, South Korea; ^5^ Department of Molecular Medicine, School of Medicine, Gachon University, Incheon, South Korea

**Keywords:** paclitaxel, metastasis, Snail, epithelial-to-mesenchymal transition (EMT), transforming growth factor-β, (TGF-β)

## Abstract

Distant relapse after chemotherapy is an important clinical issue for treating breast cancer patients and results from the development of cancer stem-like cells (CSCs) during chemotherapy. Here we report that blocking epithelial-to-mesenchymal transition (EMT) suppresses paclitaxel-induced CSCs properties by using a MDA-MB-231-xenografted mice model (*in vivo*), and breast cancer cell lines (*in vitro*). Paclitaxel, one of the cytotoxic taxane-drugs such as docetaxel, increases mesenchymal markers (Vimentin and Fibronectin) and decreases an epithelial marker (Zo-1). Blocking TGF-β signaling with the TGF-β type I receptor kinase (ALK5) inhibitor, EW-7197, suppresses paclitaxel-induced EMT and CSC properties such as mammosphere-forming efficiency (MSFE), aldehyde dehydrogenase (ALDH) activity, CD44^+^/CD24^−^ ratio, and pluripotency regulators (Oct4, Nanog, Klf4, Myc, and Sox2). The combinatorial treatment of EW-7197 improves the therapeutic effect of paclitaxel by decreasing the lung metastasis and increasing the survival time *in vivo*. We confirmed that Snail is increased by paclitaxel-induced intracellular reactive oxygen species (ROS) and EW-7197 suppresses the paclitaxel-induced Snail and EMT by attenuating paclitaxel-induced intracellular ROS. Knock-down of *SNAI1* suppresses paclitaxel-induced EMT and CSC properties. These data together suggest that blocking the Snail-induced EMT with the ALK5 inhibitor attenuates metastasis after paclitaxel-therapy and that this combinatorial approach could prove useful in treating breast cancer.

## INTRODUCTION

Breast cancer is a major health threat to women worldwide and the leading cause of cancer-related death in women [1-3]. Although progression of therapies and early detection technologies has helped the survival of cancer patients, mortality is still high due to cancer recurrence and drug resistance [4]. Since metastatic relapse is the major cause of death in cancer patients, it is important to understand the mechanism of the metastatic relapse after chemotherapy.

The acquisition of metastatic potential during breast carcinogenesis and during chemotherapy is in part due to the epithelial-to-mesenchymal transition (EMT). During EMT, cancer cells go through the morphologic and phenotypic changes; increase in motility, dissemination, and dedifferentiation according to the signaling pathways that reprogram the gene expressions. Even though EMT participates in diverse biological processes (embryogenesis (type 1), wound healing (type 2), and cancer metastasis (type 3)), some of the underlying genetic and molecular mechanisms may be related if not identical [5, 6]. The majority of signaling pathways and various transcription factors, such as the Snail family, Twist, Zeb respond to these signals and function as master controllers of the EMT program [7]. Transforming growth factor (TGF-β) signaling is thought to be the chief inducer of EMT as it plays dual roles in tumorigenesis [8, 9]. Indeed, TGF-β acts as tumor suppressor during the early stage of tumorigenesis, but ultimately promotes [5, 10]. These actions are mediated by canonical TGF-β pathwas that include ALK-5 phosphorylation of Smad2/3 (p-Smad2/3) [11, 12].

The TGF-β signaling pathway during EMT also involves the complex formation of Snail with Smad3/Smad4 [13] as well as the regulation of the EMT master regulators, Slug, and Twist [14], which accelerate cancer metastasis [15]. Moreover, ectopic expression of Snail or Twist or exposure to TGF-β induces EMT in immortalized human mammary epithelial cells (HMLE) and increases the formation of tumor spheres and CD44^+^/ CD24^−^ cells, also known as cancer stem-like cells (CSCs) [16, 17]. The subset of tumor cell population have CSC properties, such as slow replication and cancer drug resistance, providing an explanation to the development of tumor recurrence and drug resistance [18, 19]. CSCs are capable of self-renewal and multi-lineage differentiation as key tumor-initiating cells and more resistant to anti-cancer therapies such as chemotherapy, hormone therapy, and radiotherapy than the majority of more differentiated cancer cells, showing correlation with the poor prognosis in cancer patients [20-25]. These studies together suggest a direct link between EMT and the achievement of CSC properties, which may be required for metastasis following chemotherapy. Furthermore, TGF-β signaling is increased in chemo-resistant cancer [26-30] and it is a major driving force behind breast cancer EMT, development of CSCs and metastasis [21]. In this study, we demonstrate that blocking Snail-induced EMT with a TGF-β inhibitor, EW-7197, can inhibit CSC development and that the combinatorial use of EW-7197 with paclitaxel is a superior strategy for preventing metastasis.

## RESULTS

### Paclitaxel-resistance is related to the mesenchymal traits

**Figure 1:**
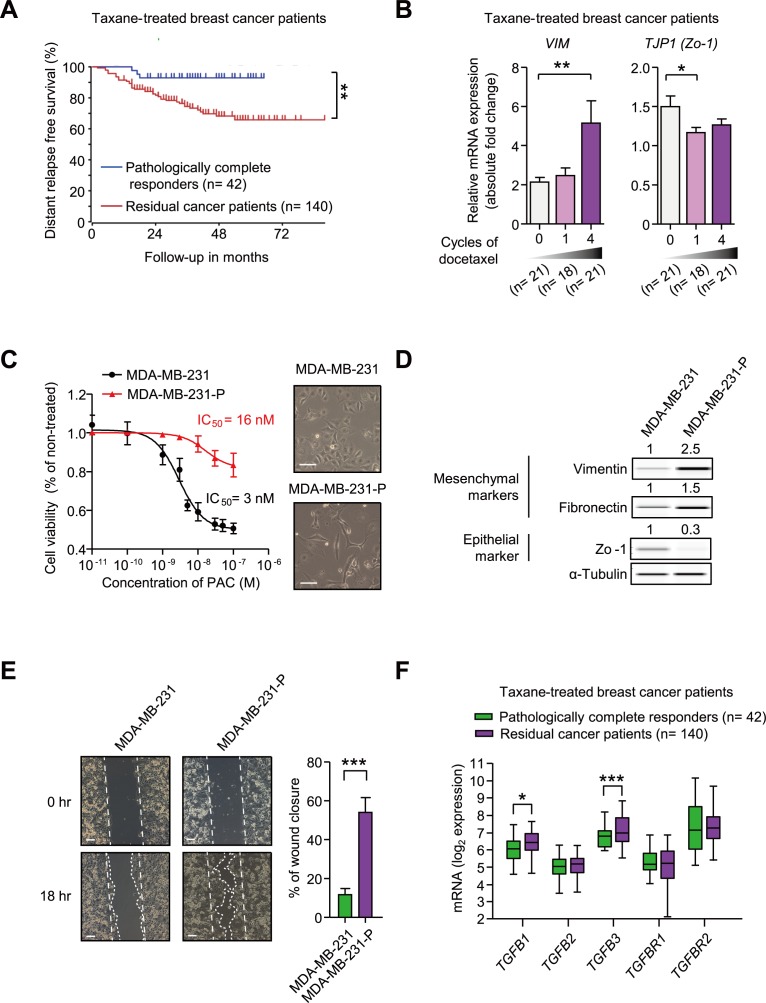
**A.** A Kaplan-Meier plot of the distant relapse-free survival based on responses to taxane in breast cancer patients, Booser dataset from ‘R2: Genomics Analysis and Visualization Platform (http://r2.amc.nl)’. A total 162 patients were classified into the pathologically complete responders (*n* = 42) and the residual cancer patients (*n* = 140) after the taxane treatment. The significance was calculated by a log-rank test. **B.** mRNA expression levels of *VIM* and *TJP1* in breast cancer patients from the Korde dataset from Oncomine (www.oncomine.com) (*n* = 21, 18, 21, at 0-, 1-, 4-cycle of docetaxel, respectively). **C**.-**E**. The comparison of MDA-MB-231 and the paclitaxel-resistant MDA-MB-231 cells (MDA-MB-231-P) in terms of IC_50_ values of paclitaxel and cell morphology **C.**, EMT markers **D.**, and motility **E.**. **C.** IC_50_ values of paclitaxel were analyzed by cell viability assays and right panels show the images of the phase-contrast microscopic images of both cells (× 100, scale bar: 100 μm). **D.** The protein expression of EMT markers was analyzed by WES analysis. The values above each lane indicate the relative intensity of bands as normalized by the intensity of α-Tubulin. **E.** The motilities of cells were measured by the wound healing assay. The left panels show the phase-contrast microscopy images (× 100, scale bar: 100 μm) at the beginning of the experiment (0 hour) and the end point (18 hour). The right graph shows the percentage of wound closure as mean ± SD (*n* = 3). **F.** The mRNA expression of the ligands or receptors of TGF-β signaling in breast cancer patients, Booser dataset from ‘R2: Genomics Analysis and Visualization Platform (http://r2.amc.nl)’. The statistical values were calculated by student's t-test (between two groups) or ANOVA with Dunnett's multiple comparison test (among groups more than three). *, **, and *** indicate *P* < 0.05, *P* < 0.01, and *P* < 0.005, respectively).

We analyzed clinical data in an attempt to better understand the features of taxane-resistant breast cancer. In Booser cohort, breast cancer patients received pre-operative (neoadjuvant) taxane-treatment. After the treatment, patients were divided into two responder groups according to pathologic response (pathologic complete response or residual cancer burden). In Figure 1A, the Kaplan-Meier plot for distant relapse-free survival shows that taxane-resistant cancers were more likely to relapse in distant regions, even though they had been removed by surgical operation. The result signifies that it is important to prevent the metastatic relapse of taxane-resistant cancers to accomplish valid remedy in breast cancer patients. Since EMT is known to be closely related to the metastatic potential of breast cancer [31], we analyzed the changes of EMT markers in breast cancers following taxane treatment. In Figure 1B, the mRNA expression of Vimentin (*VIM*), a mesenchymal marker, was continuously increased following the treatment of docetaxel, but Zo-1 (*TJP1*), an epithelial marker, was decreased. We therefore examined if paclitaxel-resistant MDA-MB-231 (MDA-MB-231-P) cells also had higher levels of EMT markers.

As shown in Figure 1C, MDA-MB-231-P had an IC_50_ of 16 nM for paclitaxel, whereas MDA-MB-231 had an IC_50_ of 3 nM. MDA-MB-231-P cells are resistant to cytotoxic effect of 3 nM paclitaxel based on cell viability assays (Figure 1C) and cell cycle analysis (Supplementary Figure 1A). Moreover, the morphology of MDA-MB-231-P cells had changed into a more spindle shape. In accordance with the morphological changes, the expression of the mesenchymal proteins, Vimentin and Fibronectin, showed 2.5-fold and 1.5-fold increases, respectively, whereas the expression of the epithelial protein, Zo-1, showed a 0.3-fold decrease in MDA-MB-231-P cells when compared to those of MDA-MB-231 cells (Figure 1D). We compared the motility of the MDA-MB-231-P cells with that of the MDA-MB-231 cells using wound healing assays (Figure 1E). The percentage of wound closure was significantly increased in the MDA-MB-231-P cells by 4.6 fold compared to that of MDA-MB-231 cells showing the similar growth rate as that of the parental MDA-MB-231 cells in paclitaxel-free media (Supplementary Figure 1B). These results suggest that the mesenchymal traits are correlated with taxane-resistance in patients as well as in cells *in vitro*. We subsequently examined whether TGF-β, a major driver of EMT, is related to taxane-resistance in breast cancer patients. Figure 1F showed that the expression of TGF-β1 and TGF-β3 ligands were significantly higher in taxane-resistant patients than those of responsive patients. These data suggest that taxane-resistant cancer is associated with mesenchymal-traits and that TGF-β ligands are increased in taxane-resistant patients.

### TGF-β inhibitor suppresses the paclitaxel-induced mesenchymal traits in MDA-MB-231-xenografted mice

**Figure 2:**
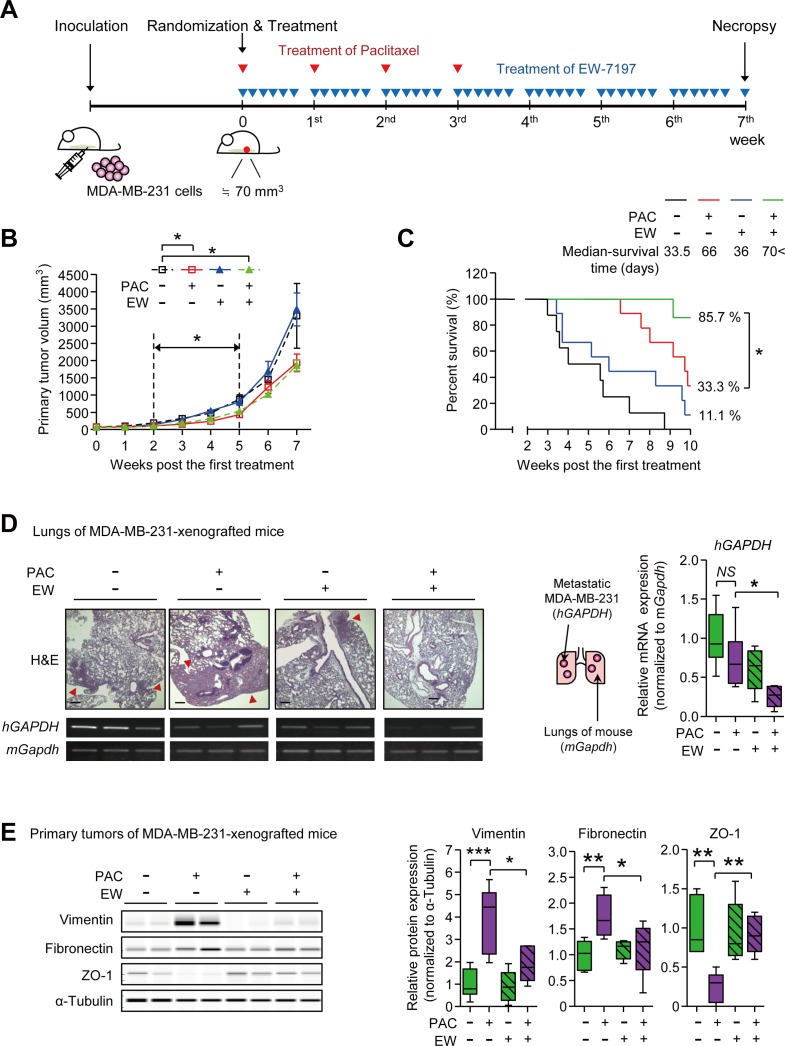
**A.** The schematic of the experimental breast cancer mouse model for the combinatorial treatment of EW-7197 and paclitaxel (MDA-MB-231-xenografted mice). Mice were inoculated with MDA-MB-231 cells and the mice, of which the tumor sizes were around 70 mm^3^, were randomly grouped and the treatment started as described in the Material and Methods section. Mice were treated with paclitaxel once a week for a total 4 cycles and EW-7197 was treated for 5 consecutive days a week for 7 weeks (for efficacy test) or 10 weeks (for survival test). (B-E) Analyses of tumor growth and metastasis in MDA-MB-231-xenografted mice **B.** The primary tumor volume was calculated once a week by the formula: volume (mm^3^) = (length (mm)) × (width (mm))^2^ × 0.5 (*n* = 5~6/group). **C.** The Kaplan-Meier analysis of survival (*n* = 6~7/group) **B**. and **C**. Black line or black dotted line represents the control group, red line represents the paclitaxel-treated group, blue line represents the EW-7197-treated group, and green line represents the combinatorial EW-7197 and paclitaxel-treated group. **D.** The representative phase-contrast microscopy images of H&E-stained lungs and the mRNA expressions of human *GAPDH* (*hGAPDH*) and mouse *Gapdh* (*mGapdh*) in lungs for analyzing the metastasis to lungs (*n* = 5~6/group). **E.** The protein expression of EMT markers in primary tumors of the MDA-MB-231-xenografted mice (*n* = 5~6/ group). Lysates were obtained directly from primary tumor tissues. In the box and whisker plots, boxes display the median values with upper and lower quartiles, and whiskers show the ranges. The statistical values were calculated by ANOVA with Dunnett's multiple comparison test (* indicates *P* < 0.05 and *NS* means no significance).

We established the MDA-MB-231-xenograft NOD.CB17-Prkdc^scid^ (NOD) mouse model to examine if a TGF-β inhibitor would inhibit paclitaxel-induced EMT (Figure 2A). The mRNA expression level of *TGFB1* was increased by paclitaxel *in vivo* as previously reported [30] (Supplementary Figure 2A). The treatment of paclitaxel reduced the cancer burden starting from the 2nd week (after 2 cycles of paclitaxel) until the 5th week (Figure 2B and Supplementary Figure 2B). During this period, the TGF-β inhibitor, EW-7197 could not reduce primary cancer burden in alone treatment and the combinatorial EW-7197 treatment could not enhance the cytotoxic effect of paclitaxel (Figure 2B). Notably, EW-7197 synergistically prolonged the survival time (Figure 2C). As paclitaxel reduced the burden of the primary tumor, it dramatically prolonged the median-survival time to 66 days, whereas that of the control group was 33.5 days. However, the survival of the paclitaxel-group decreased rapidly once the first death started. Even though the effect of treatment with EW-7197 alone on survival was minimal (the median survival time = 36 days), the combinatorial treatment of EW-7197 with paclitaxel extended the survival time over that of paclitaxel alone (Figure 2C).

We then examined the effect of EW-7197 on metastasis as EW-7197 improved the survival time without affecting the size of tumor burden. MDA-MB-231 cells metastasized to the lungs of mice, as identified by H&E staining and expression of the human *GAPDH* gene (Figure 2D). We confirmed the species specificity of primer sets used in this RT-PCR experiment (Supplementary Figure 2C). As shown in Figure 2D, paclitaxel did not have a statistically significant effect on lung metastasis when compared to the control group, even though paclitaxel reduced the cancer burden efficiently. Notably, the combinatorial treatment of EW-7197 with paclitaxel significantly decreased the lung metastasis by 0.27-fold when compared to the paclitaxel, which decreased the lung metastasis by 0.73-fold. Protein analysis of the primary tumors of MDA-MB-231-xenografted mice revealed that EW-7197 inhibited EMT in paclitaxel-treated cancers (Figure 2E and Supplementary Figure 2D). The protein expression levels of Vimentin and Fibronectin, two mesenchymal markers, were increased by 3.86- and 1.74-fold, respectively, in paclitaxel-treated primary tumors. At the same time, the protein expression of Zo-1, an epithelial marker, was decreased to 0.24-fold. Combinatorial treatment of EW-7197 and paclitaxel ameliorated the paclitaxel-induced increase of Vimentin and Fibronectin and restored the paclitaxel-decreased Zo-1 levels *in vivo* (Figure 2E). These results suggest that pacliataxel treatment increases the metastatic potential in breast cancer by enhancing mesenchymal traits *in vivo*, and EW-7197 inhibits the post-therapy-increase of metastatic potential by attenuating the paclitaxel-induced enhancement of mesenchymal traits.

### Paclitaxel induces mesenchymal traits *via* Snail

**Figure 3:**
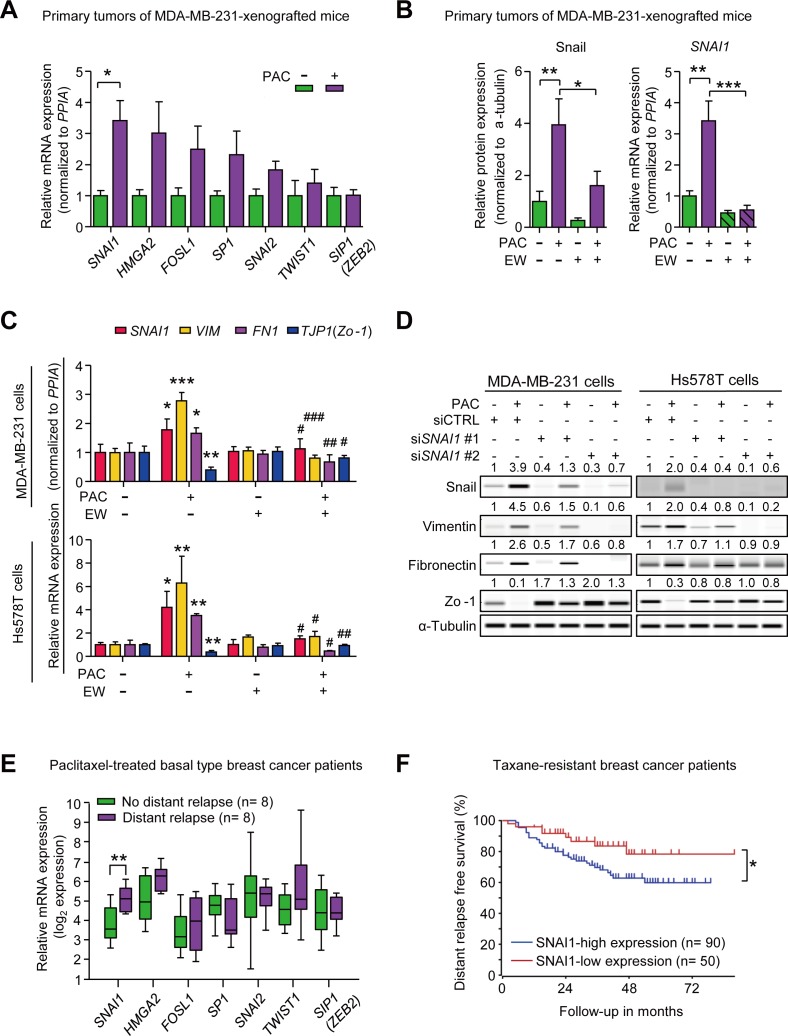
**A.** The relative mRNA expression of EMT-transcriptional factors in MDA-MB-231-xenografted mice (*n* = 5~6/group). **B.** The relative protein and mRNA level of Snail (*SNAI1*) in the primary tumors of MDA-MB-231-xenografted mice (*n* = 5~6/group). (A and B) Lysates were obtained directly from primary tumor tissues. **C.** The relative mRNA expression of *SNAI1*, *VIM*, *FN1*, and *TJP1* (*ZO-1*) in MDA-MB-231 cells and Hs578T cells after 24-hour-treatment of paclitaxel (3 nM) with or without EW-7197 (100 nM) (*n* = 3/group). *, **, and *** indicate *P* < 0.05, *P* < 0.01, and *P* < 0.005, respectively, vs the non-treated control group. ^#^, ^##^, and ^###^ indicate *P* < 0.05, *P* < 0.01, and *P* < 0.005, respectively, vs the paclitaxel-treated group. **D.** The protein expression of Snail and EMT markers in Snail-knocked-down MDA-MB-231 or Hs578T cells after 24-hour-treatment of paclitaxel (3 nM). The values above each lane represent the relative intensity of bands as normalized by the intensity of α-tubulin. **E.** The relative mRNA expression of EMT-transcriptional factors in breast cancer patients who had residual cancer burden after the paclitaxel-treatment. Patients had basal type breast cancer and were classified according to the prognosis after taxane-therapy, no distant relapse (*n* = 8) and distant relapse (*n* = 8) (Booser dataset from ‘R2: Genomics Analysis and Visualization Platform (http://r2.amc.nl)’) **F.** Kaplan-Meier analysis of distant relapse-free survival according to the *SNAI1* expression in taxane-resistant breast cancer patients (Booser dataset from ‘R2: Genomics Analysis and Visualization Platform (http://r2.amc.nl)’). Lower 50^th^ value of *SNAI1* expression was set as a cut-off. Bar graphs display the means ± SE (*in vivo*) or SD (*in vitro*). In the box and whisker plots, boxes display the median values with upper and lower quartiles, and whiskers show the ranges. The statistical values were calculated by student-t-test (between 2 groups) or ANOVA with Dunnett's multiple comparison test (among groups more than 3). Log-rank test was used in Kaplan-Meier analysis (*, **, and *** indicate *P* < 0.05, *P* < 0.01, and *P* < 0.005, respectively).

To investigate the underlying mechanism of the paclitaxel-induced mesenchymal traits, we tried to identify the EMT-regulators that enhanced mesenchymal traits in response to paclitaxel. To begin with, we confirmed that paclitaxel-induced mesenchymal traits were correlated with the increased TGF-β signaling by detecting the phosphorylation of Smad2 and Smad3 (p-Smad2/3) *in vivo* (primary tumors of paclitaxel-treated MDA-MB-231-xenografted mice) and *in vitro*. In Supplementary Figure 3A, paclitaxel increased p-Smad2/3 and combinatorial treatment of EW-7197 decreased p-Smad2/3 both *in vivo* and i*n vitro* (MDA-MB-231 cells). Then, we compared the mRNA levels of several TGF-β-related EMT-regulators including *SNAI1*, *SNAI2*, *HMGA2*, *FOSL1*, *SP1*, *ZEB1*, *ZEB2* (*SIP1*), and *TWIST1* [8, 32, 33] in primary tumor tissues of MDA-MB-231-xenografted mice. Although expression of many EMT-regulators appeared to increase with paclitaxel, only changes in *SNAI1* were statistically significant (Figure 3A). This result was confirmed by Wes analysis of primary tumor tissues in MDA-MB-231-xenografted mice. Paclitaxel increased Snail protein levels by 3.95-fold, which subsequently decreased significantly with the combinatorial treatment of EW-7197 (Figure 3B and Supplementary Figure 3B). Furthermore, EW-7197 inhibited the paclitaxel-induced transcriptional activation of *SNAI1* (Figure 3B).

To clarify the effect of paclitaxel and EW-7197 on Snail, we conducted *in vitro* assays in various breast cancer cell lines including luminal and basal types. Cells were treated with IC_50_ concentrations of paclitaxel for 24 hours. The protein levels of Snail were analyzed by Wes analysis (Supplementary Figure 3C and 3D). In luminal breast cancer cell lines such as T47D and ZR75B cells, paclitaxel slightly increased the expression of Snail by 1.3- and 1.5-fold, respectively. On the other hand, in basal breast cancer cell lines such as MDA-MB-468, Hs578T, and MDA-MB-231 cells, paclitaxel increased Snail by paclitaxel-induced increases of Snail were higher than those of luminal breast cancer cells by 1.9-, 2.8-, and 3.9-fold, respectively. Among tested breast cancer cell lines, paclitaxel-induced increases of Snail were the highest in MDA-MB-231 and Hs578T cells, which are highly invasive breast cancer cell lines, associated with cancer stem-like features (CD44^+^/CD24^−^) [34-36]. Therefore, we conducted the advanced *in vitro* experiments using both MDA-MB-231 and Hs578T cell lines. In accordance with the *in vivo* results, the treatment with the IC_50_ concentration of paclitaxel increased the expression of *SNAI1*, *VIM* and *FN1*, and decreased *TJP1* (*Zo-1*) expression, which was abolished by the concomitant treatment of 100 nM EW-7197 (Figure 3C and Supplementary Figure 3B). Treatment with EW-7197 alone did not decrease the basal level of *SNAI1* expression in both cell lines. The effect of paclitaxel on Snail was confirmed by knocking-down *SNAI1* with siRNAs in both MDA-MB-231 and Hs578T cells. As shown in Figure 3D and Supplementary Figure 2C, siRNAs targeting *SNAI1* (si*SNAI1*) suppressed the effects of paclitaxel on Snail, Vimentin, Fibronectin, and Zo-1 and si*SNAI1* #2 appeared to be more effective than si*SNAI1* #1. Both si*SNAI1* #1 and si*SNAI1* #2 decreased the basal expression of Snail in MDA-MB-231 as well as in Hs578T cells. These results suggest that Snail is the major EMT-regulator that confers cancer cells mesenchymal traits in response to paclitaxel *in vitro*. We also analyzed clinical data in order to look for the major EMT-transcriptional factors which would drive metastasis in taxane-resistant basal breast cancer patients (Booser dataset from ‘R2: Genomics Analysis and Visualization Platform (http://r2.amc.nl) (Figure 3E). We compared the expression of EMT-transcriptional factors between relapse (*n* = 8) and non-relapse (*n* = 8) groups who had taxane-resistant basal breast cancer and discovered that only expression levels of *SNAI1* were significantly different. More importantly, the higher expression of *SNAI1* is related to the distant metastatic relapse in patients who had taxane-resistant breast cancer (Booser dataset from ‘R2: Genomics Analysis and Visualization Platform (http://r2.amc.nl)’) (Figure 3F). Based on these experimental and clinical data, it appears that Snail is an important EMT-transcriptional factor that drives the metastatic relapse after taxane-therapy.

### Paclitaxel induces Snail *via* reactive oxygen species

**Figure 4:**
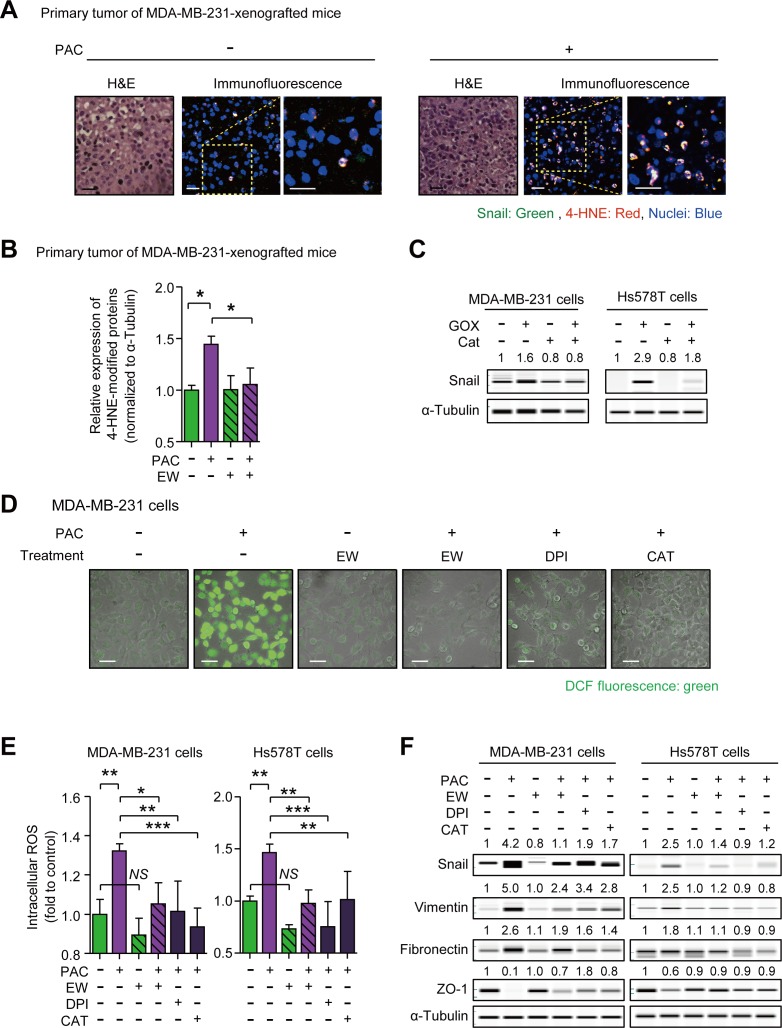
**A.** Co-localization of Snail and 4-hydroxynonenal (4-HNE), one of the indicators of reactive oxygen species (ROS) stress, were visualized by an immunofluorescence assay in the primary tumors of MDA-MB-231-xenografted mice. In the con-focal images, green fluorescence indicates Snail and red fluorescence indicates 4-HNE (× 400 or × 800, scale bar: 20 μm). **B.** The relative quantification of the 4-HNE-modified proteins in the primary tumor of MDA-MB-231-xenografted mice (*n* = 5~6/group) by western blots. **C.** The protein level of Snail in MDA-MB-231 and Hs578T cells after the 8-hour-treatment of glucose oxidase (GOX) (7.5 mU/ml) with or without catalase (Cat) (500 U/ml), ROS scavenger. (D and E) DCF-sensitive intracellular ROS were measured in paclitaxel-treated MDA-MB-231 or Hs578T cells with or without the pre-treatment of EW-7197 (100 nM), diphenyleniodonium (DPI) (100 nM), and Cat (500 U/ml) **D.** by a con-focal microscopy (× 200, scale bar: 50 μm) and **E.** by a micro-plate reader (described in Supplementary Material and Methods). **F.** The protein level of Snail and EMT markers in MDA-MB-231 or Hs578T cells after 24-hour-treatment of paclitaxel with or without EW-7197 (100 nM), DPI (100 nM), Cat (500 U/ml). In WES analysis, the values above each lane mean the relative intensity of bands as normalized by the intensity of α-Tubulin. Bar graphs show means ± SE (*in vivo*) or SD (*in vitro*). The statistical values were calculated by ANOVA with Dunnett's multiple comparison test (*, **, and *** indicate *P* < 0.05, *P* < 0.01, and *P* < 0.005, respectively. *NS* means no significance).

In order to gain the insight into the mechanism of Snail-induction by paclitaxel and the inhibitory effect of EW-7197, we visualized the localization of ROS stress and Snail by measuring 4-hydroxynonenal (4-HNE) as a marker of ROS stress. The results showed the co-localization of Snail with 4-HNE in primary tumors of paclitaxel-treated mice (Figure 4A). Moreover, western blot analysis confirmed that paclitaxel increased the 4-HNE-modification of proteins, and that concomitant treatment with EW-7197 aborted the increase of 4-HNE (Figure 4B and Supplementary Figure 4A). To investigate the role of ROS on the expression of Snail, we conducted an *in vitro* assay with MDA-MB-231 and Hs578T cells. We treated glucose oxidase (GOX) to cells with and without catalase (CAT), a ROS scavenger, and as shown in Figure 4C, GOX increased the protein level of Snail by 1.6- and 2.9-fold in MDA-MB-231 and Hs578T cells, respectively, while catalase ameliorated the GOX effects. These data show that ROS stress can be an inducer of Snail.

To investigate the effect of EW-7197 on paclitaxel-induced ROS, we conducted *in vitro* experiments. In Figure 4D and 4E, intracellular ROS was increased by 1.32- and 1.46-fold in paclitaxel-treated MDA-MB-231 and Hs578T cells, respectively. An NADPH oxidase (NOX) inhibitor, diphenyleneiodonium chloride (DPI), and a ROS scavenger, CAT were used to clarify the effect of paclitaxel-induced ROS on EMT. NOX is responsible for paclitaxel-induced ROS production by converting oxygen (O_2_) into superoxide (O_2_^•−^) and by releasing superoxide outside cells. The relatively unstable superoxide is subsequently dismutated into hydrogen peroxide (H_2_O_2_), which is membrane-permeable and can increase intracellular ROS [37]. DPI, CAT, and EW-7197 reduced the paclitaxel-induced increases of intracellular ROS in MDA-MB-231 and Hs578T cells. EW-7197 also inhibited paclitaxel-induced intracellular ROS *in vitro*, in agreement with the *in vivo* study (Figure 4B, 4D and 4E). We next investigated the role of ROS on paclitaxel-induced Snail and mesenchymal markers and as shown in Figure 4F, Paclitaxel increased Snail, Vimentin, and Fibronectin, whereas it decreased Zo-1 in both cells. Notably, EW-7197, DPI, and CAT, all of which decreased paclitaxel-induced intracellular ROS, attenuated the increase of Snail and at the same time ameliorated the up-regulation of Vimentin and Fibronectin, and restored Zo-1 in both cell lines (Figure 4F). These results suggest that the increased intracellular ROS mediates the paclitaxel-induced increase of Snail and mesenchymal traits and in addition, that the inhibitory effect of EW-7197 on Snail is related to reducing intracellular ROS, which is increased by paclitaxel.

### Paclitaxel induces cancer stem-like properties *via* Snail

**Figure 5:**
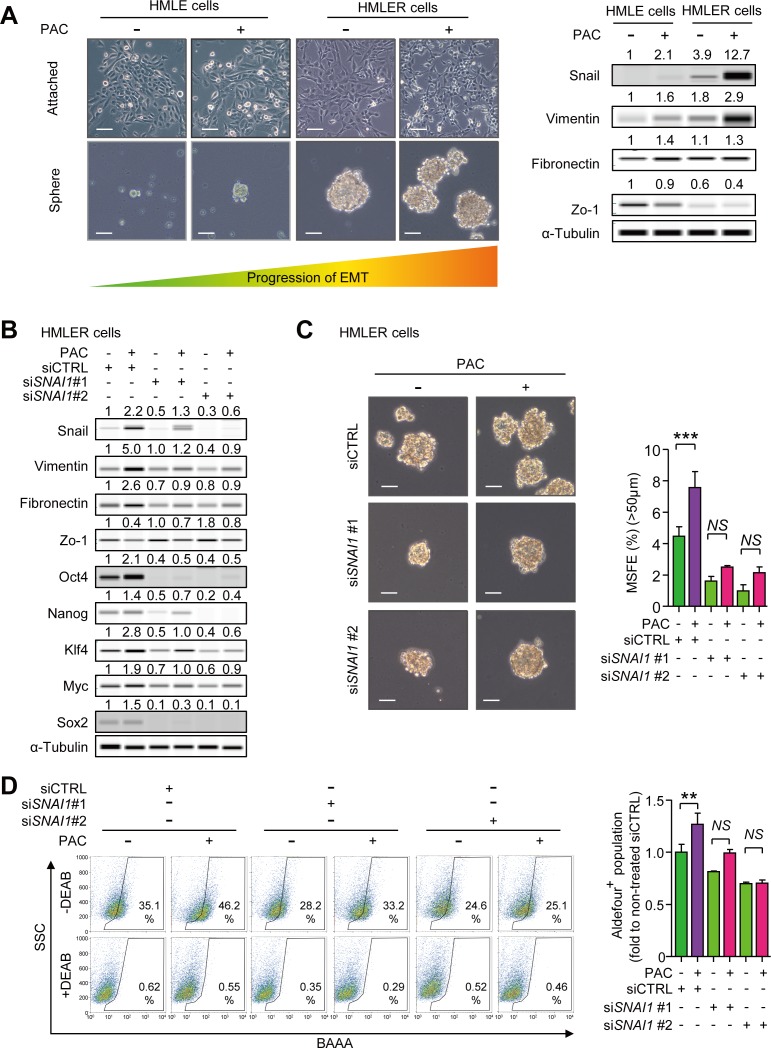
**A.** HMLE and HMLER cells were treated with the IC_50_ concentrations of paclitaxel for 24 hours in the attached-culture condition and the protein level of Snail and EMT markers were detected by WES analysis. With these cells, the abilities of mammosphere-formation were confirmed by five-day-culture in the ultra-low attachment condition. The left-upper panels show the representative microscopic images (× 100, scale bar: 50 μm) and the right panels show the protein levels of Snail and EMT markers after 24-hour-treatment in attached culture. The left-lower panels show the representative microscopic images of spheres after five-day-culture in the ultra-low-attachment condition (× 100, scale bar: 50 μm). **B.** The protein level of Snail and EMT markers in Snail-knocked-down HMLER cells after the 24-hour-treament of paclitaxel (4 nM) in the attached-culture condition. The values above each lane mean the relative intensity of bands as normalized by the intensity of α-tubulin. **C.** The mammosphere forming efficiency (MSFE) was measured by five-day-culture in the ultra-low attachment condition in the Snail-knocked-down HMLER cells which were treated with paclitaxel (4 nM) as above. The left panel show the phase-contrast microscopic images of spheres (× 100, scale bar: 50 μm) after the five-day-culture in the ultra-low attachment condition and the graph shows MSFE over 50 μm (*n* = 3/group). **D.** The activity of ALDH in Snail-knocked-down HMLER cells was analyzed by Aldeflour^TM^ assay, after the 24-hour-treatment of paclitaxel (4 nM) as above. The left panel shows the representative 2D-plots of Aldeflour^TM^ assay with or without DEAB (N, N-diethylaminobenzaldehyde) and the graph shows the ALDH-positive population as folds to the mean value of the non-treated control group (*n* = 3/group). **C**. and **D**. Bar graphs show means ± SD. The statistical values were calculated by ANOVA with Dunnett's multiple comparison test (*, **, and *** indicates *P* < 0.05, *P* < 0.01, and *P* < 0.005, respectively).

There have been reports showing that EMT generates cancer-stem like traits in breast epithelial cells [16,17]. We investigated whether paclitaxel enhances cancer stem-like properties inducing mesenchymal traits in HMLE and HMLER cells. At first, we calculated the IC_50_ values of paclitaxel in HMLE and HMLER cells by cell viability assays (Supplementary Figure 5A and 5B). We then treated both cell lines with the IC_50_ concentrations of paclitaxel for 24 hours and investigated the changes of morphology and EMT markers. In Figure 5A, HMLER cells showed an elongated and disseminated morphology than that of HMLE cells and the treatment of paclitaxel changed the morphologies of both HMLE and HMLER cells into a spindle shape in culture. Moreover, the expression of Snail and Vimentin were higher in HMLER than HMLE cells and paclitaxel increased Snail and Vimentin in both cell types. The expression of Zo-1 was lower in HMLER than HMLE cells and paclitaxel decreased Zo-1 in both. Although there was no significant difference in Fibronectin expression between HMLE and HMLER cells, paclitaxel slightly increased the expression of Fibronectin in both cell lines. Furthermore, Paclitaxel increased the sphere-forming ability in both HMLE and HMLER cells along with the enhanced mesenchymal markers (Figure 5A and Supplementary Figure 5C). HMLE cells, which lack sphere-forming ability on their own, could form spheres in ultra-low attachment culture after exposure to paclitaxel, although the growth of spheres did not reach a 50 μm cutoff. HMLER cells expressing the mesenchymal markers could form a number of spheres, and paclitaxel increased the sphere forming ability. From these results, we concluded that enhancement of mesenchymal traits was associated with acquiring sphere-forming ability.

Because the sphere-forming ability of HMLE cells was not sufficient for measuring sphere-forming efficiency, we conducted siRNA experiments only in HMLER cells to determine the role of Snail in paclitaxel-induced stem-like properties. In Figure 5B, knocking-down *SNAI1* diminished the paclitaxel-induced increase of Snail, Vimentin and Fibronectin and restored levels of Zo-1. In accordance with these EMT markers, pluripotent stem cell regulators including Oct4, Nanog, Klf4, Myc (c-Myc), and Sox2, were increased by paclitaxel and *SNAI1*-knock down aborted the increase of theses regulators (Figure 5B). In Figure 5C, paclitaxel-induced mesenchymal traits and pluripotency regulators affected the self-renewal function of cancer stem-like cells. Paclitaxel also increased mammosphere-forming efficiency (MSFE) by 1.8-fold and knock-down of *SNAI1* attenuated the paclitaxel-induced increase of MSFE. Furthermore, the activity of aldehyde dehydrogenase (ALDH), a marker of cancer stem-cells, was increased by paclitaxel in HMLER cells by 1.27-fold and this effect was lost with knock-down of *SNAI1* (Figure 5D). These results suggest that paclitaxel-induced mesenchymal traits enhance the stem-like properties and are mediated at least in part by Snail.

### TGF-β inhibition reduces cancer stem-like properties induced by paclitaxel and Snail

**Figure 6:**
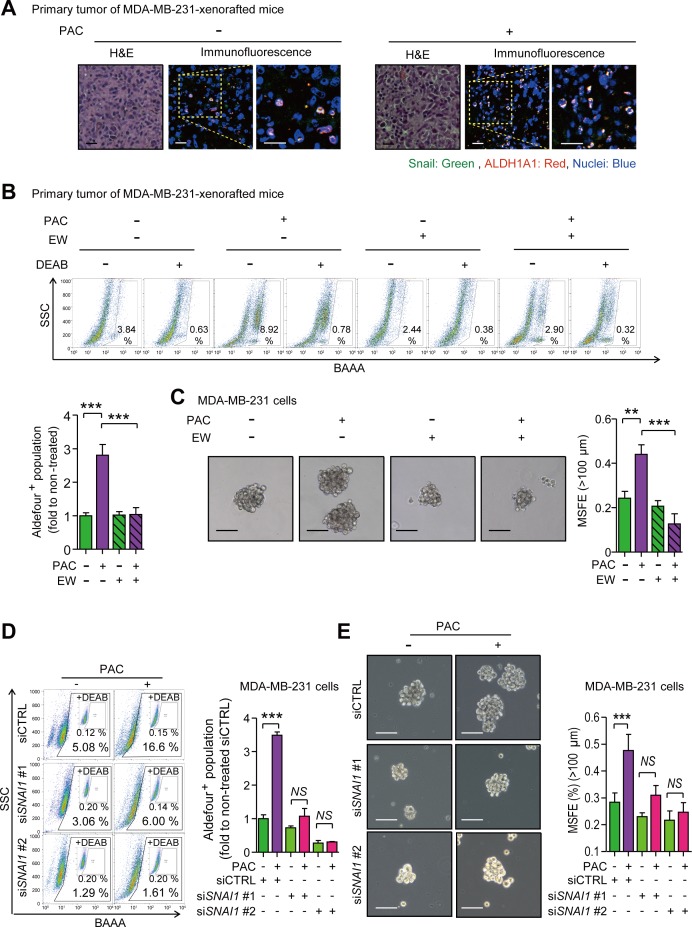
**A.** Co-localization of Snail and ALDH1A1 was visualized by immunofluorescence assay in the primary tumors of MDA-MB-231-xenografted mice. In the confocal images, green fluorescence indicates Snail and red fluorescence indicates ALDH1A1 (× 400 or × 800, scale bar: 20 μm). **B.** The ALDH activity in the primary tumors of the MDA-MB-231-xenografted mice was analyzed with an Aldeflour^TM^ assay. Upper panels show the representative 2D-plots of Aldeflour^TM^ assay with or without DEAB (N, N-diethylaminobenzaldehyde), an inhibitor of ALDH, and graphs shows the ALDH-positive population as the fold to the mean value of the non-treated control group (*n* = 5~6/group). **C.** Sphere forming efficiency was calculated after the five-day-culturing MDA-MB-231 cells in the ultra-low attachment plates which were treated by paclitaxel (3 nM) with or without EW-7197 (100 nM) for 24 hours in attached-culture condition. The left panels show the phase-contrast microscopy images of spheres (× 100, scale bar: 100 μm) and the graph shows the mammosphere-forming efficiency (MSFE) over 100 μm (*n* = 3/group). **D.** The activity of ALDH in Snail-knocked-down MDA-MB-231 cells was measured with an Aldeflour^TM^ assay after the treatment of paclitaxel or EW-7197. The control siRNA (siCTRL) or *SNAI1* siRNA (si*SNAI1*) were treated in MDA-MB-231 cells and paclitaxel was treated as the same condition as above. The left panels show the representative 2D-plots of Aldeflour^TM^ assay with or without DEAB and the right graph shows the ALDH-positive population as fold to the mean value of the control group (*n* = 3/group). **E.** MSFE was performed after the treatment of paclitaxel in Snail-knocked-down cells. The experimental process was same above. The left panels show the phase-contrast microscopy images of spheres (× 100, scale bar: 100 μm) and the right graph shows MSFE over 100 μm (*n* = 3/group). Bar graphs show means ± SD (*in vitro*) and SE (*in vivo*). The statistical values were calculated by student-t-test (between two groups) or ANOVA with Dunnett's multiple comparison test (among groups more than three) (** and *** indicate *P* < 0.01 and *P* < 0.005, respectively).

As Snail plays a key role in enhancing mesenchymal traits and stem-like properties in response to paclitaxel, we sought to confirm whether Snail was associated with stem-like properties by visualizing the localization of Snail and ALDH1A1, the surface marker of cancer stem cells in many types of cancer. Both were indeed co-localized in primary tumors of MDA-MB-231-xenografted mice (Figure 6A). We then analyzed the activity of ALDH in primary tumors of MDA-MB-231-xenografted mice and determined that paclitaxel significantly increased the activity of ALDH by 2.81-fold, which was ameliorated by combinatorial treatment with EW-7197, although EW-7197 alone had no effect (Figure 6B). In accordance with *in vivo* data, paclitaxel induced ALDH activity and the co-treatment of EW-7197 ameliorated the effect of paclitaxel in MDA-MB-231 and Hs578T cells (Supplementary Figure 6A). Another marker of stem-like cancer cells, CD44^high^/CD24^low^, was analyzed in both cells. The cancer stem-like population was increased by paclitaxel and co-treatment with EW-7197 abolished this effect (Supplementary Figure 6B). We conducted a functional assay of stem-like cells by measuring mammosphere forming efficiency (MSFE). Paclitaxel increased MSFE and the co-treatment of EW-7917 decreased the effect of paclitaxel in MDA-MB-231 cells (Figure 6C) and Hs578T cells (Supplementary Figure 6C). To determine whether these stem-like properties induced by paclitaxel were mediated by Snail, we depleted *SNAI1* using siRNA. *SNAI1*-knocked down cells were treated with paclitaxel with or without EW-7197 for 24 hours and ALDH activity was then quantified. Paclitaxel increased ALDH activity 3.4-fold while conversely, this effect was interrupted by *SNAI1* depletion (Figure 6D). Furthermore, the paclitaxel-induced increase of MSFE was also attenuated by knocking-down *SNAI1* (Figure 6E), which together suggests that EW-7197 inhibits the cancer stem-like properties that are induced by paclitaxel and mediated by Snail.

### EW-7197 inhibits the pluripotency regulators that are induced by paclitaxel and Snail

**Figure 7:**
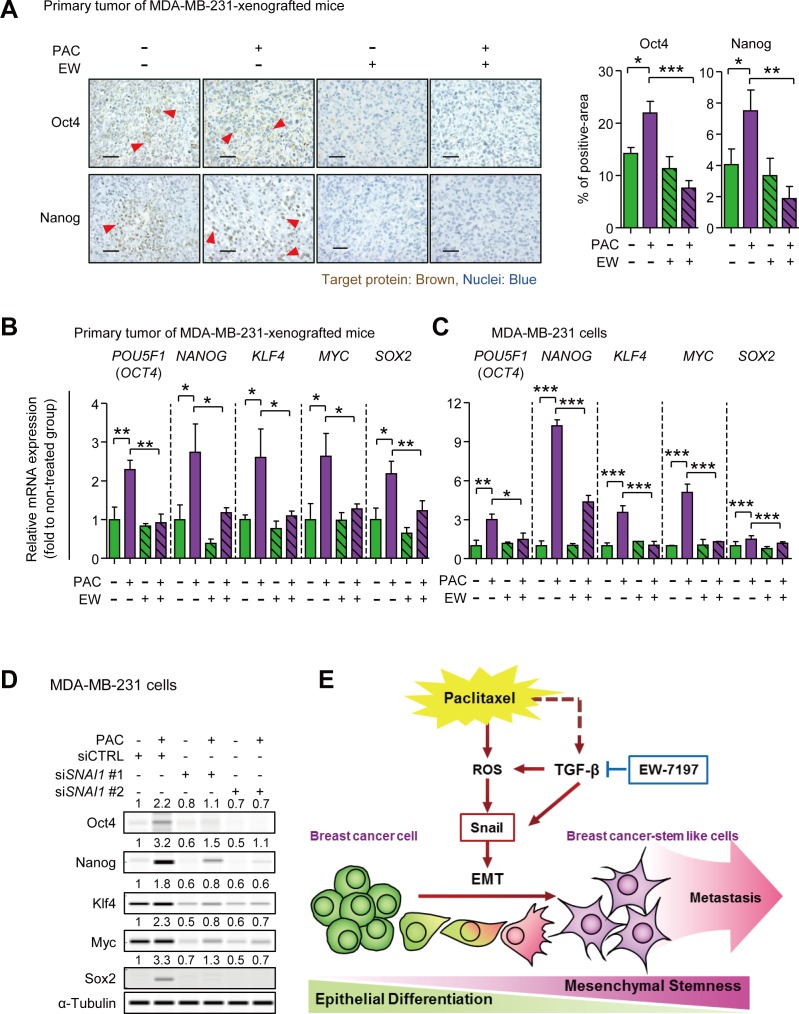
**A.** Immunohistochemistry assays (IHC) for Oct4 and Nanog in primary tumors of MDA-MB-231-xenografted mice. The left panels show the representative phase-contrast images of IHC for Oct4 or Nanog (× 400, scale bar: 50 μm), and the right graphs show the target-protein-positive area as percent of the field, quantified by the Image J program (*n* = 5~6/group). **B.** The relative mRNA expressions of the pluripotency regulators, Oct4, Nanog, Klf4, c-Myc (Myc), and Sox2, were analyzed in primary tumors of MDA-MB-231-xenografted mice by qRT-PCR (*n* = 5~6/group). **C.** The relative mRNA expressions of pluripotency regulators in MDA-MB-231 cells after 24-hour-treatment of paclitaxel (3 nM) with or without EW-1797 (100 nM). **D.** The protein level of pluripotency regulators in control or Snail-knocked down MDA-MB-231 cells after 24-hour-treatment of paclitaxel (3 nM). The values above each lane represent the relative intensity of bands as normalized by the intensity of α-Tubulin. Bar graphs show means ± SD (*in vitro*) and SE (*in vivo*). The statistical values were calculated by ANOVA with Dunnett's multiple comparison test (*, ** and *** indicate *P* < 0.05, *P* < 0.01 and *P* < 0.005, respectively). **E.** Summary plot of paclitaxel-induced EMT and CSCs. TGF-β is known to be secreted in paclitaxel-treated breast cancer (dotted line). The TGF-β inhibitor, EW-7197, reduces TGF-β-induced Snail and also inhibits the reactive oxygen species (ROS), which are generated by paclitaxel. ROS induces Snail driving EMT in breast cancer cells. The mesenchymal breast cancer cells show cancer stem-like cell (CSC) properties and lead to distant metastasis *in vivo*. EW-7197 blocks a Snail induction by paclitaxel and reduces CSC properties showing inhibitory effect on distant metastasis.

We investigated the effect of EW-7197 on the paclitaxel-induced pluripotency regulators that play a critical role in self-renewal of cancer stem cells. To begin with, we found that Klf4 was continuously increased following docetaxel treatment in breast cancer patients (Korde datasets from Oncomine) (Supplementary Figure 7A). In Figure 7A, based on immunohistochemistry assay, Oct4 and Nanog were increased in paclitaxel-treated tumors of MDA-MB-231-xenografted mice, and decreased by co-treatment with EW-7197. Furthermore, according to qRT-PCR, the mRNA expressions of *POU5F1* (*OCT4*), *NANOG*, *KLF4*, *MYC* (*c-MYC*), and *SOX2* showed 2.29-, 2.74-, 2.60-, 2.64-, and 2.18-fold-increases, respectively, in paclitaxel-treated tumors of MDA-MB-231-xenografted mice, and these changes were abolished by the co-treatment with EW-7197 (Figure 7B). In accordance with the *in vivo* data, similar phenomena were reproduced *in vitro* using MDA-MB-231 cells. The 24-hour-treatment of paclitaxel increased the mRNA expression of *POU5F1* (*OCT4*), *NANOG*, *KLF4*, *c-MYC*, and *SOX2* by 3.00-, 10.22-, 3.55, 5.09, and 1.51-fold to that of the control, respectively, whereas co-treatment with EW-7197 decreased expression (Figure 7C). These results suggest that EW-7197 inhibits paclitaxel-induced cancer stem-like properties *in vivo* and *in vitro* by inhibiting the pluripotency regulators. In Figure 7D, paclitaxel-induced up-regualtion of the pluripotency regulators was diminished with knocked-down of *SNAI1.* Paclitaxel induced protein expression of Oct4, Nanog, Klf4, Myc (c-Myc) and Sox2 by 2.2-, 3.2-, 1.8-, 2.3-, and 3.3-fold to that of control in siCTRL treated cells and in addition, Snail knock-down reduced the basal expression of Oct4, Nanog, Klf4, Myc (c-Myc) and Sox2. Inhibiting Snail expression also diminished paclitaxel-induced increases in the pluripotency regulators. Based on these results, we suggest that EW-7197 can inhibit the paclitaxel-induced up-regulation of pluripotency regulators *via* Snail.

## DISCUSSION

A number of studies have also shown that mesenchymal traits are enhanced during long-term paclitaxel exposure in breast, ovarian and prostate cancer cells [38-40]. Additionally, development of invasiveness and resistance to doxorubicin or tamoxifen is acquired by EMT process in MCF7cells [41, 42]. In accordance with the previous studies, we show that MDA-MB-231 cells undergo EMT by paclitaxel *in vitro* (Figure 1) and *in vivo* (Figure 2). Also, primary breast cancer tissues of patients express EMT following administration of taxanes and more vulnerable to metastatic relapse (Figure 1A and 1B). Based on these results together, we investigated the therapeutic effect of EMT inhibition to prevent the metastatic relapse following chemotherapy.

Tumor microenvironment consists of various cellular components such as immune cells, fibroblasts and vascular cells, which all together can favor tumor's growth and metastatic progression by producing various inflammatory cytokines including TGF-β, resulting in the silence of anti-cancer immune responses [43]. Previously, we reported that treatment of EW-7197 as a single agent inhibits breast cancer metastasis by blocking EMT and by increasing immune surveillance *via* cytotoxic T cells (CTLs) in the immunocompetent syngeneic mouse models such as 4T1-allografted mouse and mouse mammary tumor virus (MMTV)/-cNeu mouse models [44]. In the current study, combinatorial treatment of EW-7197 efficiently inhibits lung metastasis which cannot be blocked by paclitaxel in MDA-MB-231-xenografted NOD.CB17-Prkdc^scid^ mice. Since functional T cells and B cells are absent in NOD.CB17-Prkdc^scid^ mice, we could not investigate the effects of paclitaxel and EW-7197 on CTL-mediated immune surveillance in this model. For the same reason, therapeutic effect of EW-7197-alone-treatment was more potent in immunocompetent syngeneic mouse models [44] than in an immunodeficient xenograft mouse model of the current study. However, the existence of functional B cells and T cells seems to be insignificant for paclitaxel to stimulate TGF-β signaling in tumor microenvironment, based on the results in Supplementary Figures 2A and 3A.

In this study, we showed that EMT is induced by paclitaxel in primary tumors of MDA-MB-231-xenografted mice and that co-treatment of EW-7197 efficiently blocked paclitaxel-induced EMT based on the protein analysis (Figure 2E). Even though paclitaxel directly induces EMT in cancer cells *in vitro* (Figure 3C), we cannot rule out the possibility that stromal cells in tumor microenvironment may contribute to the changes of EMT markers *in vivo* [45].

We further demonstrate that Snail is the main EMT-transcriptional factor that mediates paclitaxel-induced EMT and CSC properties. Interestingly, basal-type (triple negative) breast cancer cell lines are more readily capable of enhancing Snail expression in response to paclitaxel (Supplementary Figure 3C). This result may be correlated with the microarrays of 52 widely-used breast cancer cell lines [36]. It shows that highly aggressive basal-type breast cancer cell lines are characterized by both the high expressions of mesenchymal markers including *TGFB1* and the markers associated with cancer stem-like features (CD44^+^/CD24^−^). It is quite important that paclitaxel increases Snail in aggressive breast cancer cells because the high expression of Snail indicates a poor prognosis in breast cancer patients [46, 47].

ROS are conventionally thought to be cytotoxic causing cell damage at high levels. Recently, ROS has gained intensive interest as an EMT inducer playing as second messengers in various signal transduction pathways [48]. ROS mediates the TGF-β-induced activation of mitogen-activated protein kinases (MAPK) signaling, which drives EMT [49, 50]. ROS also increased the Snail expression in Nuclear factor-κB (NF-κB) dependent manner in response to matrix metalloproteinase-3 (MMP-3) [51]. Paclitaxel increases ROS by increasing NADPH oxidase (NOX) activity [52] and in this study, we documented that paclitaxel-induced ROS increased the Snail expression and drive EMT in breast cancer cells (Figure 4). Interestingly, other microtubule-targeting drugs (docetaxel, nocodazole, and vincristine), which have been reported to increase ROS in cancer cells [52, 53], increased the intracellular ROS and EMT markers in MDA-MB-231 cells (*in vitro*) (Supplementary Figure 8A and 8B). EW-7197 reduces paclitaxel-induced ROS stress *in vivo* reducing the paclitaxel-induced ROS generation *in vitro*. We previously reported that EW-7197 inhibits NOX families and further decrease ROS stress in pathological conditions both *in vivo* and *in vitro* [54]. These studies together suggest that the increased intracellular ROS mediates the paclitaxel-induced up-regulation of Snail and mesenchymal traits, indicating that the inhibitory effect of EW-7197 on Snail-EMT may be related to reduction in intracellular ROS.

We showed that inhibiting Snail *via* blocking TGF-β signaling reduced the CSC properties induced by paclitaxel *in vivo* and *in vitro* (Figures 6 and 7). These results are agreement with previous reports showing that paclitaxel-induced Snail expression is associated with chemo- or radio-therapy resistance by enhancing CSCs and distant metastasis [38, 40, 55, 56]. Knock-down of Snail decreased the activity of ALDH and the expressions of Sox2, Nanog, Oct4, pluripotency regulators, in pancreatic cancer cells [57]. Notably, Klf4 showed continuous increase according to paclitaxel administration in breast cancer patients (Supplementary Figure 7A). Klf4 is required for maintaining stem-like properties and for cell migration and invasion in breast cancer [58]. Snail-induced EMT is closely related to CSCs, hence targeting Snail is a potential therapeutic strategy for treating resistance acquired by EMT.

## MATERIALS AND METHODS

### Analysis and visualization of open source clinical data

Breast cancer clinical data were obtained and analyzed using the Booser dataset [59] from ‘R2: Genomics Analysis and Visualization Platform (http://r2.amc.nl)’ and Korde dataset [60] from ‘Oncomine (https://www.oncomine.org)’.

### Cell culture and mammosphere-forming assay

The human breast cancer cell line MDA-MB-231, MDA-MB-468, ZR75B, and T47D were obtained from the ATCC. Hs578T was obtained from the Korean Cell Line Bank (Seoul, Korea), and the immortalized human breast epithelial cells (HMLE) and Ras-transformed HMLE (HMLER) cells from Dr. Robert A. Weinberg (Whitehead Institute for Biomedical Research, Cambridge, Massachusetts, USA). No authentication of these cell lines was performed by the authors, with the exception of performing a mycoplasma test using the e-MycoTM plus mycoplasma PCR detection kit (iNtRON Biotechnology, Korea) once a year. All cells were maintained as described previously [44]. MDA-MB-231, MDA-MB-468, and Hs578T cells were grown in RPMI (Gibco Laboratories, Grand Island, NY) complemented with 5% HI-FBS. ZR75B and T47D cells were grown in RPMI with 10% FBS. HMLE and HMLER cells were grown in DMEM/F-12 (1:1) (Gendepot, CA, USA) complemented with 10% HI-FBS, 10 μg/ml insulin (Sigma, St. Louis, MO), 10 ng/ml EGF (Sigma, St. Louis, MO), and 0.5 μg/ml hydrocortisone (Sigma, St. Louis, MO). For measuring mammosphere-forming efficiency (MSFE), cells were seeded in ultra-low attachment dishes for 4 days in DMEM/F-12 (1:1) supplemented with B27, 20 ng/ml FGF (Invitrogen, Carlsbad, CA, USA), and 20 ng/ml EGF (Sigma, St. Louis, MO). After five days, the number of spheres was counted. MSFE indicates the number of spheres divided by the original number of cells seeded and presented as%.

### RNA extraction and real-time quantitative RT-PCR (qRT-PCR)

Transcript levels of various genes were quantified in cells under different conditions or in mouse tissues using quantitative reverse transcriptase-polymerase chain reaction (qRT-PCR) assays as described previously [44], and with primers listed in Supplementary Table 1.

### Establishment of paclitaxel-resistant MDA-MB-231 cells

Paclitaxel-resistant MDA-MB-231 cells were established by sub-culturing the survived cells at 3 nM of paclitaxel in paclitaxel in RPMI media (5% HI-FBS). The cells became resistant to the growth inhibitory effect of paclitaxel showing the similar growth rate as that of the parental cells. We confirmed the acquired-resistance to paclitaxel by cell cycle analysis using propidium Iodide (PI) staining and IC_50_ concentrations were compared using cell viability assays. The detailed cell cycle analysis and cell viability assays are described in the Supplementary Materials and Methods section.

### Wound healing assay

Cells were seeded in culture inserts (Ibidi, GmbH, Martinsried, Germany) in 5% HI-FBS media. Inserts were removed and media were replaced to serum-reduced media (0.2% HI-FBS) (time = 0). Cells were incubated for 18 hours (end point). Phase-contrast images of cells were captured with a camera attached to a microscope (Carl Zeiss, Oberkochen, Germany). The wound area at zero time or the end point was measured using the ImageJ (NIH, Bethesda, MD, USA). The area of wound closure was calculated as a percentage of the initial wound area.

### Experimental breast cancer mouse model for combinatorial treatment of EW-7197 with paclitaxel

All animal experimental procedures were conducted in accordance with protocols preapproved by the Institutional Animal Care and Use Committee (IACUC) of Ewha Womans University. Animal housing, food, and watering were maintained as described previously [44]. A total of 2×10^6^ MDA-MB-231 cells were implanted into the left #4 mammary fat pads of 6-week-old NOD.CB17-Prkdc^scid^ female mice purchased from Central Lab Animal Inc (Seoul, South Korea). When the tumor volume was approximately 70 mm^3^, mice were randomly grouped and injected with either vehicle (Cremophor:EtOH = 1:1) or PAC (10 mg/kg) *i.p.* once a week over a total 4 cycles. The day after the first injection, vehicle (artificial gastric fluid; 2 g/L NaCl (Ducksan, Korea), 3.2 g/L Pepsin (Sigma, St. Louis, MO), 0.06 M HCl (Ducksan, Korea)) or EW (2.5 mg/kg) was given *via p.o.* for five consecutive days/week over 7 weeks (*n* = 5~6/group, in efficacy experiment) or for 10 weeks (*n* = 6~7/group, in survival experiment). Metastasis to lungs was quantified by measuring mRNA levels of human GAPDH using RT-PCR. Detailed procedure is described in Supplementary Material and Methods.

### Protein analysis by capillary electrophoresis in nano-capillaries (Wes analysis)

Cells or tissues were homogenized in RIPA buffer as described previously (24) and westerns were performed using Wes automated western blotting system (ProteinSimple, San Jose, CA) according to the manufacturer's protocols. Lysates containing 200 ng/μl of total protein were separated by electrophoresis in in nano-capillary gels. The primary antibodies used are listed in Supplementary Table 2. Quantification of peak areas and gel image reconstruction was performed using Compass software (ProteinSimple, San Jose, CA). The fluorescent standards which indicate three different molecular weights were run in every capillary and α-Tubulin was used as the loading control.

### Fluorescence-activated cell sorting (FACS) for analyzing the cancer stem-like population

Aldeflour^TM^ kit (StemCell Technologies, Vancouver, BC, Canada) was used according to the manufacturer's instructions, to analyze ALDH activity. Briefly, trypsinized single cells were incubated with ALDH substrate, biodipy-aminoacetaldehyde (BAAA), and after 30 minutes incubation at 37°C, they were washed and analyzed using a BD FACS Calibur (BD Bioscience, San Diego, CA, USA). An aliquot of each sample was incubated with the ALDH inhibitor, N, N-diethylaminobenzaldehyde (DEAB). For analyzing the CD24 and CD44 surface marekrs, cells were co-stained with FITC-conjugated anti-CD24 and PE-conjugated anti-CD44 antibodies (BD Bioscience, San Diego, CA, USA), and non-stained cells and single-stained cells were used as negative and positive controls, respectively.

### Knock down of SNAI1 using siRNAs

Cells were transfected with siRNAs against *SNAI1*, or a nonspecific negative control siRNA (Bioneer, Daejeon, Korea) in media (serum-, phenol-, antibiotics-free) with lipofectamine^TM^ 2000 (Invitrogen, Carlsbad, CA, USA) according to manufacturer's instructions. Knock-down efficiency was confirmed using qRT-PCR (for mRNA) and Wes analysis (for protein). The sequences of the siRNAs used are listed in Supplementary Table 4.

### Statistical analysis

Data are presented as mean ± standard deviation (SD) (*in vitro*) or mean ± standard error of the mean (SEM) (*in vivo*). Statistical values were defined using the student's *t*-test (between two groups) or one-way ANOVA with the Dunnett's multiple comparison test (among groups more than two). A Log-rank test was used for the Kaplan-Meier analysis. Asterisks are used to indicate statistical significance (*, **, & *** = *p* < 0.05, < 0.01, & < 0.005, respectively).

Detailed methods are described in Supplementary Material and Methods for drugs, histological assays, quantification of 4-hydroxynonenal (4-HNE)-modified proteins, and detection of intracellular ROS.

## SUPPLEMENTARY MATERIAL TABLES AND FIGURES


