# Supplementary material for: Combinatorial TGF-β attenuation with paclitaxel inhibits the epithelial-to-mesenchymal transition and breast cancer stem-like cells
Source: Oncotarget. 2015 Oct 9;6(35):37526–43. doi: 10.18632/oncotarget.6063 (PMC4741946; doi:10.18632/oncotarget.6063)
Supplement: Supplementary file 1 [file oncotarget-06-37526-s001.pdf]

Combinatorial TGF-β attenuation with paclitaxel inhibits the epithelial-to-mesenchymal transition and breast cancer stem-like cells

1. **Supplementary material**

1.1 Supplementary Material and Methods

1.1.1 Reagents

The ALK5 inhibitors, EW-7197, was synthesized by Dr. Dae-Kee Kim (Ewha Womans University, Seoul, Korea). Paclitaxel (Taxol) and docetaxel (D-1000) were obtained from LC laboratories (Woburn, MA, USA). Nocodazole and Vincristine were obtained from Sigma Aldrich (St. Louis, Missouri, USA).

1.1.2 H&E staining, Immunofluorescence, and Immunohistochemistry

Formalin-fixed and paraffin-embedded sections of primary tumors and lungs were dewaxed in an OTTIX bath (Diapath, Martinengo, Italy) and stained with hematoxylin (Sigma Aldrich, St. Louis, Missouri, USA) and eosin (Diapath, Martinengo, Italy) according to the manufacturer's instructions. Images were captured with phase-contrast microscope (Carl Zeiss, Oberkochen, Germany). For detecting Snail, 4-Hydroxynonenal (4-HNE), Aldehyde dehydrogenasenase 1A1 (ALDH1A1), and p-Smad2/3 with immunofluorescence assay, formalin-fixed and paraffin-embedded sections of primary tumors were processed through antigen-retrieval and blocking procedures as described previously [1]. Slides were incubated with a primary antibody-mixture of anti-Snail mouse IgG (Cell Signaling, Beverly, MA, USA) and anti-4-HNE rabbit IgG (Abcam, Cambridge, MA, USA), a mixture of

anti-Snail mouse IgG (Cell Signaling, Beverly, MA, USA) and anti-ALDH1A1 rabbit IgG (Abcam, Cambridge, MA, USA), or a mixture of anti-p-Smad2 rabbit IgG (Cell Signaling, Beverly, MA, USA) and anti-p-Smad3 rabbit IgG (Cell Signaling, Beverly, MA, USA). Then, a mixture of Alexa 488-conjugated anti-mouse IgG (Cell Signaling, Beverly, MA, USA) and Alexa 555-coujugated anti-rabbit IgG (Cell Signaling, Beverly, MA, USA) F(ab') fragments were used to visualize the co-localizations of Snail and 4-HNE, or Snail and ALDH1A1. Alexa 488-conjugated anti-rabbit IgG F(ab') fragments were used to visualized p-Smad2/3 proteins. Fluorescence was visualized and analyzed by an LSM 510 META laser confocal microscopy system (Carl Zeiss, Oberkochen, Germany). Target protein-positive area was calculated as the percent of the total field with ImageJ software (National Institute of Health, Bethesda, MD, USA). For *in vitro* assay, cells were seeded on poly-L-lysine coated cover glass in 6-well-plates and incubated for 24 hours for attachment. Then, cells were treated with 3 nM paclitaxel with or without 30-minute-pretreatement of EW-7197 (100 nM) for 2 hours. Cells were formalin-fixed and processed as above for visualization of p-Smad2/3. For immunohistochemistry, an avidin-biotin-peroxidase complex method using ABC kit (Vector Laboratories,Burlingame, CA, USA) was adopted on formalin-fixed and paraffin-embedded sections of primary tumors. Anti-Nanog and Anti-Oct4 (Abcam, Cambridge, MA, USA) were used as primary antibodies. HRP-conjugated anti-rabbit IgG antibodies (Santa Cruz Biotechnology, Santa Cruz, CA, USA) were used. After color reaction was developed by DAB (Vector Laboratories,Burlingame, CA, USA), sections were counterstained by hematoxylin (SigmaAldrich). Target protein-positive area was calculated as the

percentage of field with Image J program (NIH Image, Bethesda, MD, USA).

1.1.3 Western Blots for quantifying 4-hydroxynonenal (4-HNE)-modified proteins

Primary tumor tissues from the xenografted-mice were homogenized in RIPA buffer and the concentration of protein was determined as described above. Protein was denatured with SDS by boiling at 95 ℃ for 5 minutes. A total 15 μg of protein was separated by electrophoresis with a western blot kit (Bio-Rad Laboratories, Richmond, CA, USA) and transferred to a polyvinylidene difluoride (PVDF) membrane (Millipore, Bedford, MA). After blocking with 5% BSA for 30 minutes at room temperature, the membrane was incubated with the anti-4-HNE antibodies (Abcam, Cambridge, MA, USA) (1: 1000) for overnight at 4 ℃, followed by incubation with peroxidase-linked anti-rabbit IgG antibody (Santa Cruz Biotechnology, Santa Cruz, CA, USA) (1:2000) for 1 hour at room temperature. Chemi-luminescence was developed with ECL (Atto, Tokyo, Japan) and detected with a digital imaging system LAS3000 (Fujifilm, Tokyo, Japan).

1.1.4 Cell cycle analysis by propidium iodide staining

Cells were harvested and fixed in 70% ethanol for 30 minutes on ice. The ethanol-fixed cells were washed with phosphate-buffered saline (PBS) and million cells were suspended in PBS. Afterwards, cells were centrifuged and 50 μl of RNase A solution (100 μg/ml) was added to ensure that only DNA is stained. 400 μl of propidium

iodide (PI) solution was directly added and cells were incubated in PI/RNaseA solution for 30 minutes in the dark at room temperature (RT). DNA contents were analyzed using a BD FACS Calibur (BD Bioscience, San Diego, CA, USA).

1.1.5 Cell viability assays

Cells were seeded in 96 well plates and treated with indicated concentrations of paclitaxel in 1% HI-FBS medium for 48 h. The percent of viable cells were analyzed with Cell Counting Kit-8 (Dojindo, Japan) according to the manufacturers' instructions. Absorbance was measured at 450 nm with a microplate reader (Molecular Device Versa- Max, USA). The viable cells were analyzed as the percent of that of non-treated control cells. The experiment was replicated 5 times. The IC_{50} value of paclitaxel was obtained using Graphpad prism 5 with non-linear regression (curve fit) (Graphpad Software Inc., San Diego, CA, USA).

1.1.6 Detection of intracellular ROS

Intracellular accumulation of ROS was estimated using the membrane-permeable fluorescent dye 2',7'-dichlorodihydrofluorescein diacetate (H_2DCFDA) (Invitrogen, Carlsbad, California, USA), which is converted to a membrane-impermeable highly fluorescent compound, dichlorofluorescin diacetate (DCF) by intracellular esterase and ROS. For microscopic detection, cells were seeded on poly-L-lysin (Sigma Aldrich, St. Louis, Missouri, USA)-coated cover slides with complete media and incubated with serum-reduced (1% HI-FBS) media for 24 h for starvation. Then, cells were pre-incubated for 30 min with EW-7197 (100 nM), catalase (500 mU/ml), or diphenyleneiodonium chloride (DPI, 100 nM), and treated cells with paclitaxel in the

presence of H_2DCFDA (10 μM) for 30 min. Fluorescence was visualized using the LSM 510 META laser confocal microscopy system (Carl Zeiss, Germany) (excitation: 488 nm, emission: 530 nm). For quantitative detection, cells were seeded in 96-well black, clear bottom culture dishes (Corning Inc., Corning, NY), and chemicals were treated as above. Fluorescnece of DCF was detected with a Gemini EM fluorescence plate reader (Molecular Devices, Munich, Germany). The effect of docetaxel, nocodazole, and vincristine on the intracellular ROS was analyzed by a Gemini EM fluorescence plate reader as described above.

1.1.7 Analysis of lung metastasis in MDA-MB-231-xenografted mice

Metastatic MDA-MB-231 cells in lungs of xenografted-mice were analyzed by quantifying the mRNA level of human GAPDH in mouse lungs. Mice were sacrificed and the total RNA was isolated from the left lobes of lungs. cDNA was synthesized as described in the Material and methods section. The mRNA level of human GAPDH was normalized by the mRNA level of mouse GAPDH. The specificity of primers was confirmed in various cell lines and mouse tissues. Primers used for detecting human *GAPDH* were forward- CCAGGGCTGCTTTTAACTCTGGTA and reverse-AGCATCGCCCCACTTGATTTTGGA. Primers used for detecting mouse *Gapdh* were forward-ATGTGTCCGTCGTGGATCTGA and reverse-TTGAAGTCGCAG-GAGACAACC.

1.2 Supplementary References

1. Son JY, Park S-Y, Kim S-J, Lee SJ, Park S-A, Kim M-J, Kim SW, Kim D-K, Nam

J-S, Sheen YY. EW-7197, a novel ALK-5 kinase inhibitor, potently inhibits breast to lung metastasis. Molecular cancer therapeutics 2014;13(7):1704-1716.

1.3 Supplementary Tables

1.3.1 Supplementary Table 1. Primers used for qRT-PCR

Species: Human		
Gene	Forward	Reverse
FN1	GAGAATGGACCTGCAAGCCCA	GTGCAAGTGATGCGTCCGC
VIM	ACCCGCACCAACGAGAAGGT	ATTCTGCTGCTCCAGGAAGCG
TJP1 (*ZO-1*)	GGAGAGGTGTTCCGTGTTGT	GAGCGGACAAATCCTCTCTG
SNAI1	CTGGGTGCCCTCAAGATGCA	CCGGACATGGCCTTGTAGCA
SNAI2	TACCGCTGCTCCATTCCACG	CATGGGGGTCTGAAAGCTTGG
TWIST	CCTGCGCAAGATCATCCCCA	GCTGCAGCTTGCCATCTTGGA
ZEB2	CGGTATTGCCAACCCTCTGGA	TTGTTGTGCCAGGGGTGTTCC
HMGA2	CAGCCCTATCACCTCATCTC	CCATTTCCTAGGTCTGCCTC
SP1	CAGCTTCAGGCTGTTCCAAA	CTGCCAACTGACCTGTCCAT
FOSL1	GGGCCTGTGCTTGAACCTGA	TCTCCGCTGCTGCTGCTACTC
TGFB1	CCCAGCATCTGCAAAGCTCC	GTCAATGTACAGCTGCCGCA
POU5F1 (*OCT4*)	GGGCTCTCCCATGCATTCAA	CACCTTCCCTCCAACCAGTT
NANOG	TGGGATTTACAGGCGTGAGC	AAGCAAAGCCTCCCAATCCC
KLF4	ACGATCGTGGCCCCGGAAAA	CAACAACCGAAAATGCACCAGCCCCA
MYC (*c-MYC*)	GCGTCCTGGGAAGGGAGATCCGGAGC	TTGAGGGGCATCGTCGCGGGAGGCTG
SOX2	TCGGCAGACTGATTCAAATAATACAG	CCATGCAGGTTGACACCGTTG

PPIA	TGCACAGACGGTCACTCAAA	TGCCATCGCCAAGGAGTAG

1.3.2 Supplementary Table 2. Antibodies for WES analysis.

Primary Antibody			
Target	Institute	Cat#	Dilution
Fibronectin	BD Bioscience, San Diego, CA	610077	1:100
Vimentin	BD Bioscience, San Diego, CA	550513	1:100
Zo-1	Cell Signaling, Beverly, MA, USA	4206S	1:100
α-Tubulin	Cell Signaling, Beverly, MA, USA	2125S	1:100
Snail	Cell Signaling, Beverly, MA, USA	3879S	1:100
Secondary Antibody			
Anti-rabbit-HRP	ProteinSimple, Santa Clara, CA	042-206	
Anti-mouse-HRP	ProteinSimple, Santa Clara, CA	042-205	

1.3.3 Supplementary Table 3. Antibodies for immunofluorescence, Immunohistochemistry and Western blots

Primary Antibody		
Target	Institute	Cat#
4-HNE	Abcam, Cambridge, MA, USA	Ab46545
Snail	Abcam, Cambridge, MA, USA	3895S
ALDH1A1	Abcam, Cambridge, MA, USA	Ab52492
Nanog	Abcam, Cambridge, MA, USA	Ab109250
Oct4	Abcam, Cambridge, MA, USA	Ab109183

Secondary Antibody		
Anti-mouse-Alexa 488	Santa Cruz Biotechnology, Santa Cruz, CA, USA	sc-2055
Anti-rabbit-Alexa 555	Santa Cruz Biotechnology, Santa Cruz, CA, USA	sc-2004

1.3.4 Supplementary Table 4. Sequences of siRNAs

siRNAs			
Target		Sense	Anti-sense
SNAI1	#1	GCGUGGGUUUUUGUAUCCA(dTdT)	UGGAUACAAAAACCCACGC(dTdT)
	#2	GACUGUGAGUAAUGGCUGU(dTdT)	ACAGCCAUUACUCACAGUC(dTdT)
	#3	GCUGCAGGACUCUAAUCCA(dTdT)	UGGAUUAGAGUCCUGCAGC(dTdT)

Supplementary Figure 1

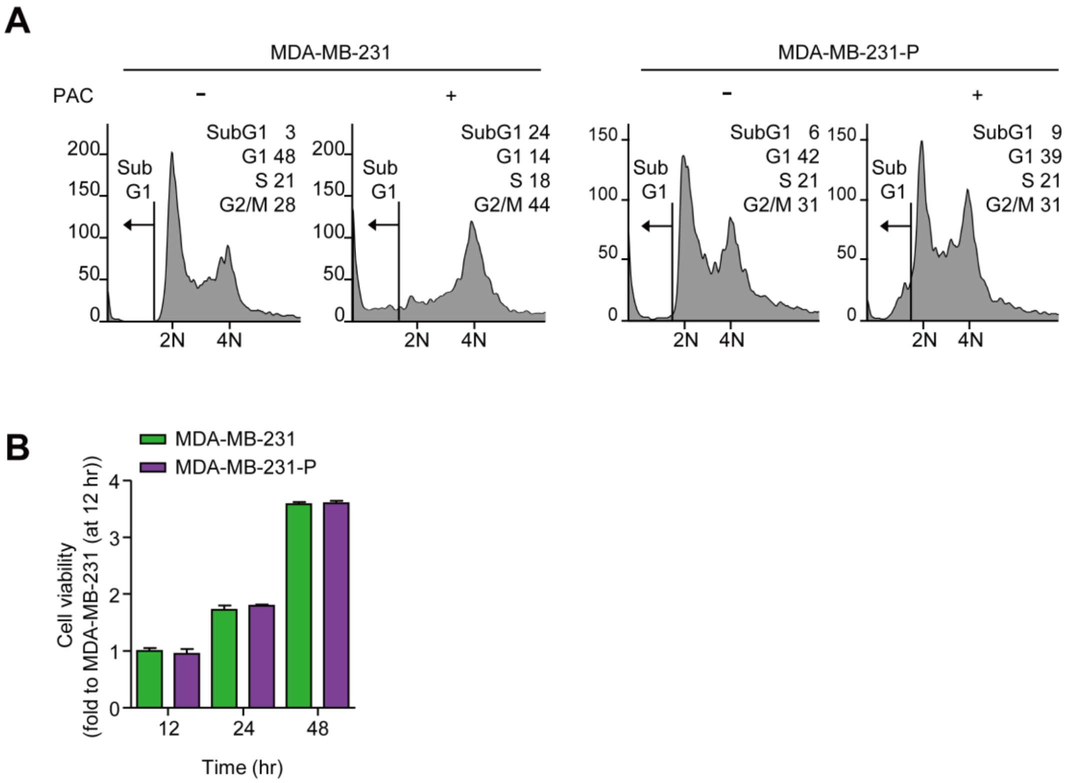

Supplementary Figure 1.

(A) Cell cycle analysis using propidium iodide (PI) was performed in MDA-MB-231 and the paclitaxel-resistant MDA-MB-231 (MDA-MB-231-P) cells. Cells were treated with 3nM paclitaxel for 24 hours in serum-reduced media (1% HI-FBS). Cells were harvested and stained with PI and analyzed using a BD FACS Calibur (BD Bioscience, San Diego, CA, USA). The histograms show the number of cells according to the DNA contents. Untreated cells were regarded as a control group. (B) The growth rates of MDA-MB-231 and MDA-MB-231-P cells were analyzed by cell viability assays using Cell Counting Kit-8 (Dojindo, Japan). Cells were seeded in 96 well plates (4000 cells/well) (0 hour) and cell viability was analyzed at various time point (12, 24, and 48 hours).

Supplementary Figure 2

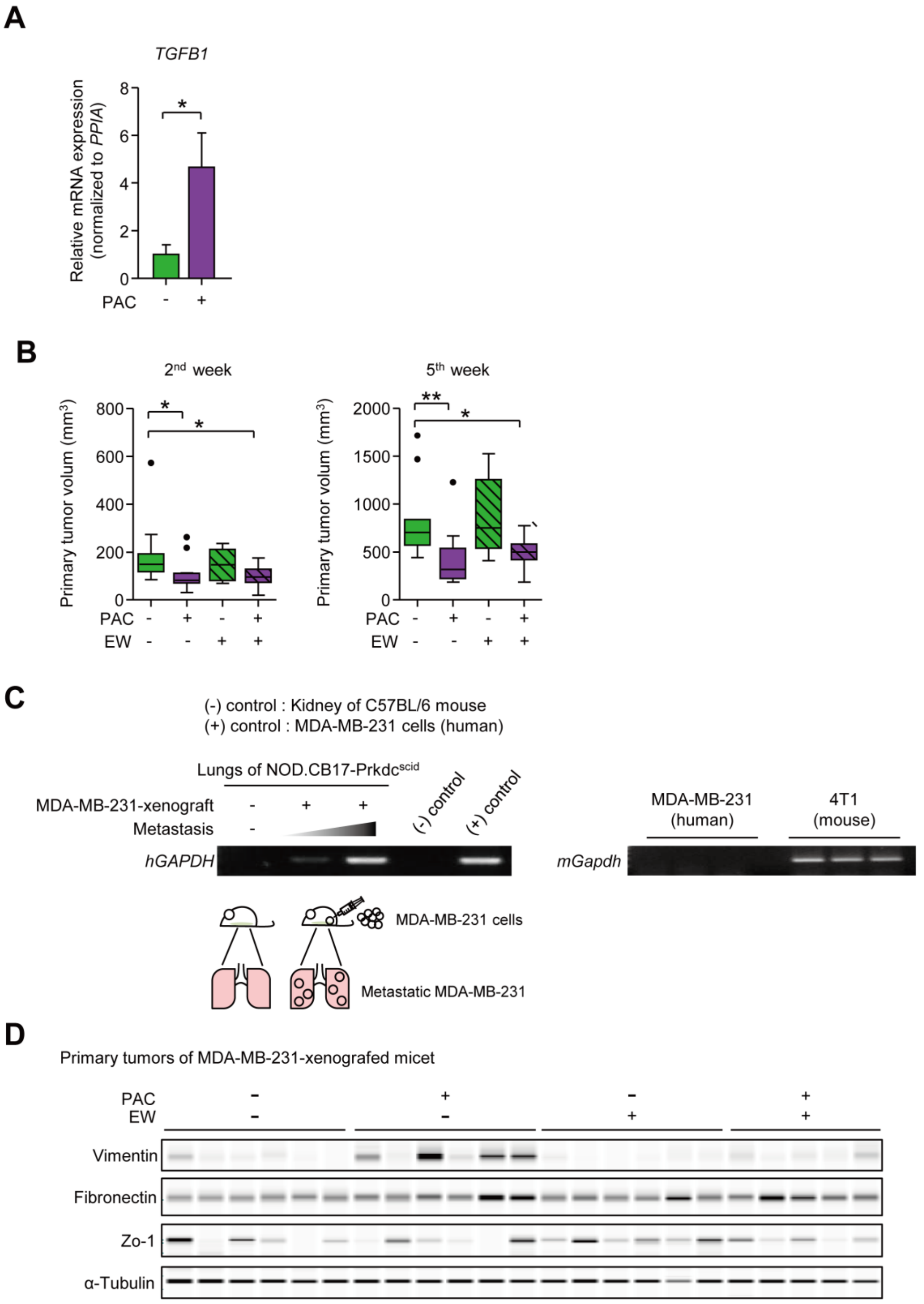

Supplementary Figure 2

(A) mRNA expression of *TGFB1* in the primary tumors of MDA-MB-231-xenografted mice. (n= 5~6/group). (B) Primary tumor volume of MDA-MB-231-xenorafted mice at 2nd or 5th week (n= 5~6/group). (C) Specificity of primer sets for detecting human GAPDH (*hGAPDH*) of the metastatic MDA-MB-231 cells in mouse lungs. Primer sets for detecting mouse Gapdh (*mGapdh*) or *hGAPDH* had no cross-reactivity. (D) Whole blot of the EMT markers in primary tumors of MDA-MB-231-xenografted mice (n= 5~6/group) (*, and ** indicate $P<0.05$, and $P<0.01$, respectively).

Supplementary Figure 3

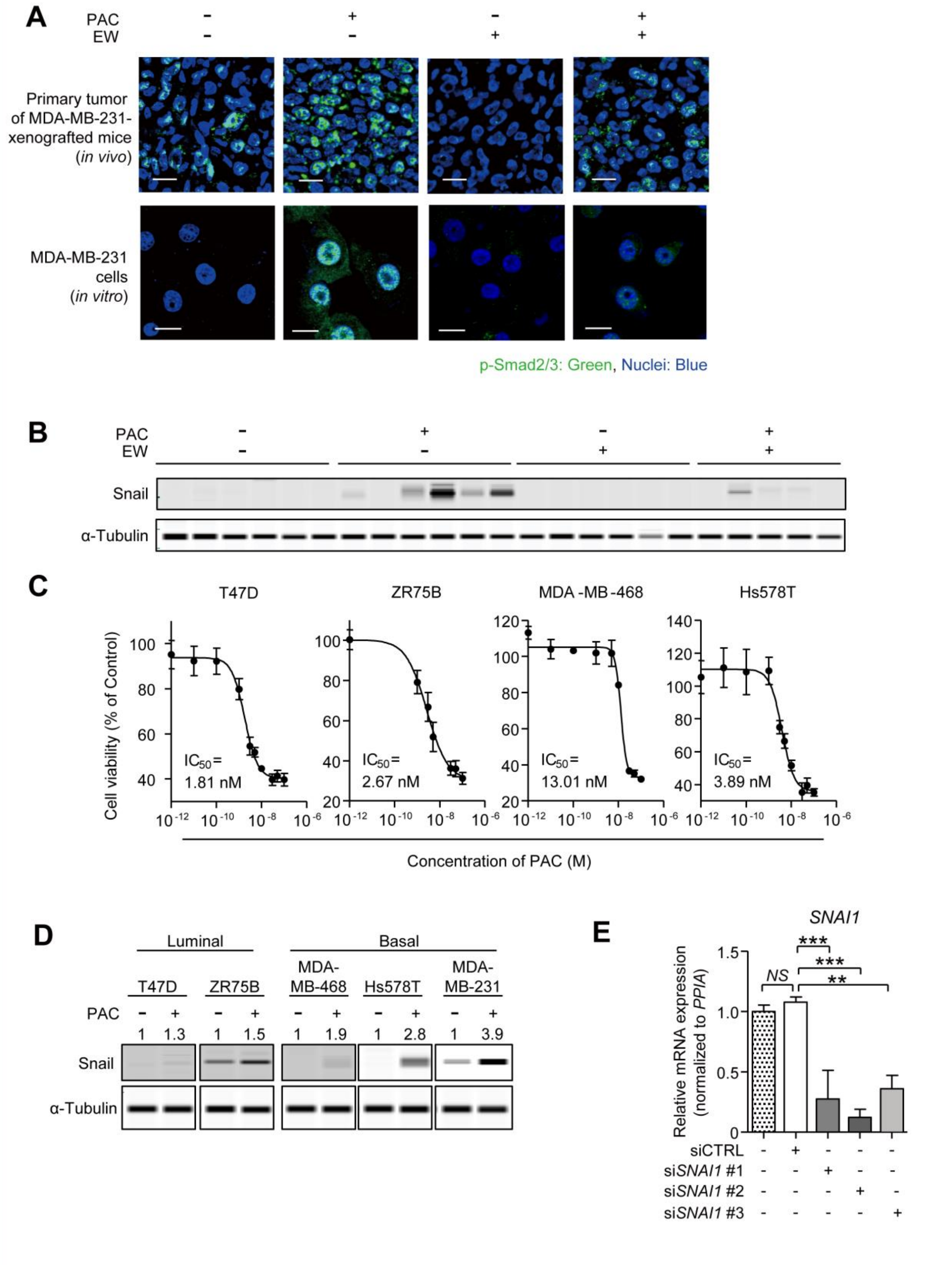

Supplementary Figure 3.

(A) Phosphorylated Smad2/3 (p-Smad2/3) in primary tumors of MDA-MB-231-xenografted mice (*in vivo*) and MDA-MB-231 cells (*in vitro*). Primary tumors of MDA-MB-231-xenografted mice (described in Material and Methods) were formalin-fixed and paraffin-embedded. MDA-MB-231 cells were treated with 3 nM paclitaxel for 2 hours with or without 30-minute-pretreatment of EW-7197 (100 nM). p-Smad2/3 proteins were visualized by an immunofluorescence assay (described in Supplementary Material and Methods). In the con-focal images, green fluorescence indicates p-Smad2/3 and blue indicates nuclei (× 400, scale bar: 20 μm). (B) Whole blot of Snail in primary tumors of MDA-MB-231-xenografted mice (n= 5~6/group) (described in Material and Methods). (C) Cell viability assay in T47D, ZR75B, MDA-MB468, and Hs578T cells in various concentrations of paclitaxel to calculate the IC_{50} values. Cells were treated with paclitaxel for 48 hours and cell viability was measured by Cell Counting Kit-8 (Dojindo, Japan) (described Supplementary Material and Methods) (D) Various breast cancer cell lines were treated with IC_{50} concentrations of paclitaxel for 24 hours in serum-reduced media (1% FBS) (1 nM for T47D, 2 nM for ZR75B, 10 nM for MDA-MB-468, 3 nM for Hs578T, and 3 nM for MDA-MB-231 cells). The protein levels of Snail were measured by Wes analysis (described in Material and Methods). The values above the lane indicate the relative intensity of bands as normalized by the intensity of α-Tubulin and as fold to control of each cell lines. (E) The mRNA expression of *SNAI1* 24 hours after the knock-down with siRNAs targeting *SNAI1*(si*SNAI1*). A non-targeting control siRNA (siCTRL) and si*SNAI1* targeting three different sequences (si*SNAI1* #1, si*SNAI1* #2, si*SNAI1* #3)

were used. The statistical values were calculated by ANOVA (** and *** indicate $P<0.01$, and $P<0.005$, respectively. *NS* means no significance).

Supplementary Figure 4

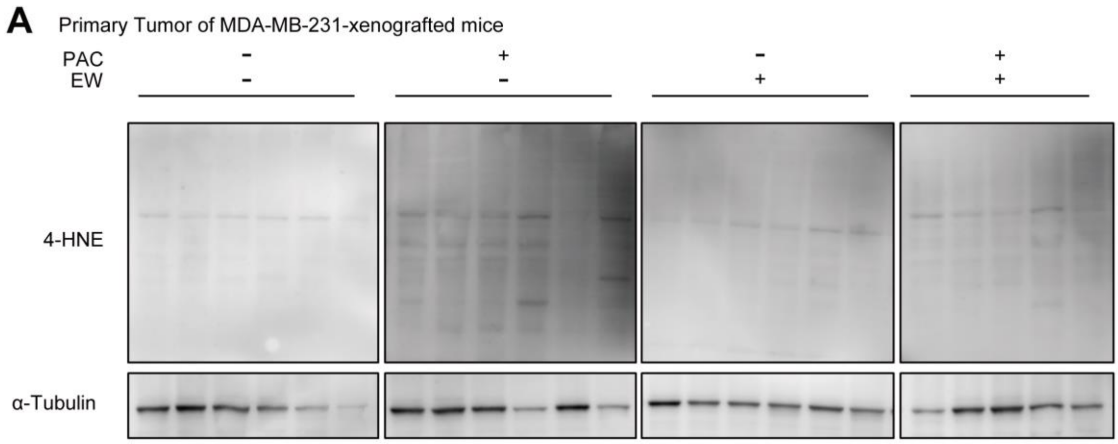

Supplementary Figure 4.

(A) Whole blot of 4-hydroxynonenal (4-HNE) in the primary tumors of MDA-MB-231-xenografted mice (n= 5~6/group).

Supplementary Figure 5

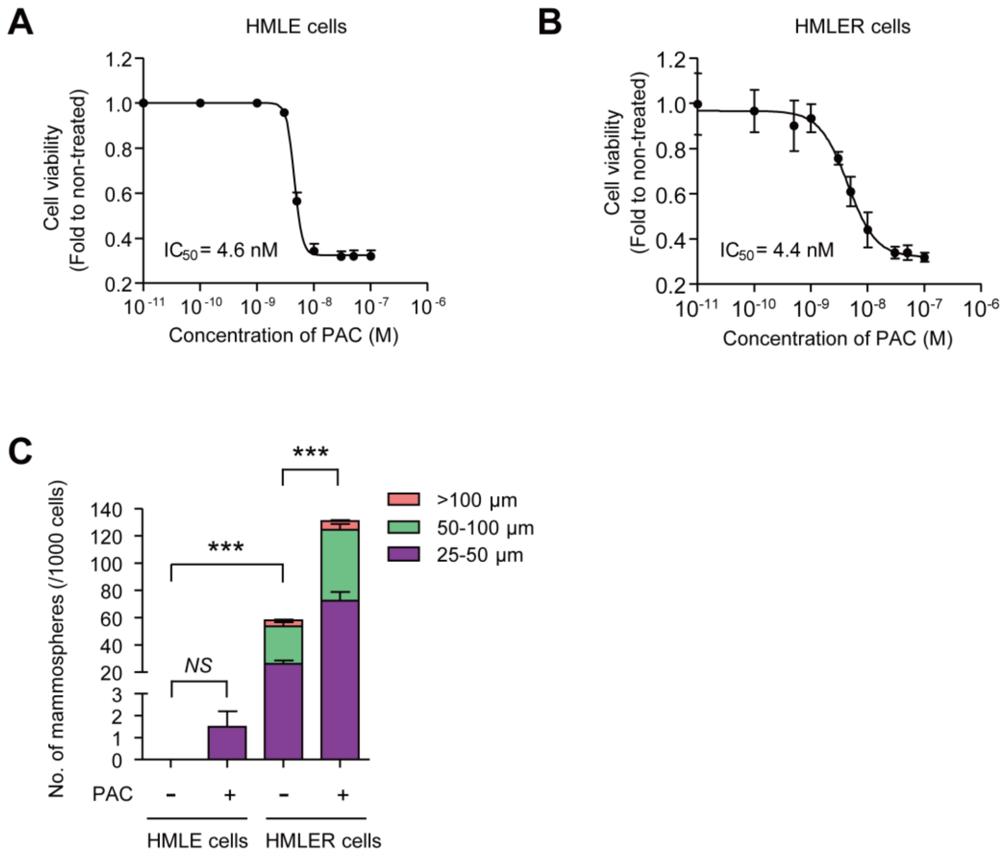

Supplementary Figure 5.

IC_{50} values of paclitaxel in HMLE (A) or HMLER cells (B) were calculated by cell viability assays with various concentrations of paclitaxel. (C) Mammosphere-froming efficiency (MSFE) was analyzed in HMLE and HMLER cells. Cells were treated with 4 nM paclitaxel for 24 hours, harvested, and seeded in ultra-low attachment wells in sphere-forming media described in Material and Methods section. After 5 days, the number of spheres was counted according to the diameter. MSFE indicates the number of spheres divided by the original number of cells seeded and presented as % (*** indicates $P<0.005$ and *NS* means no significance). Untreated cells were regarded as a control group.

Supplementary Figure 6

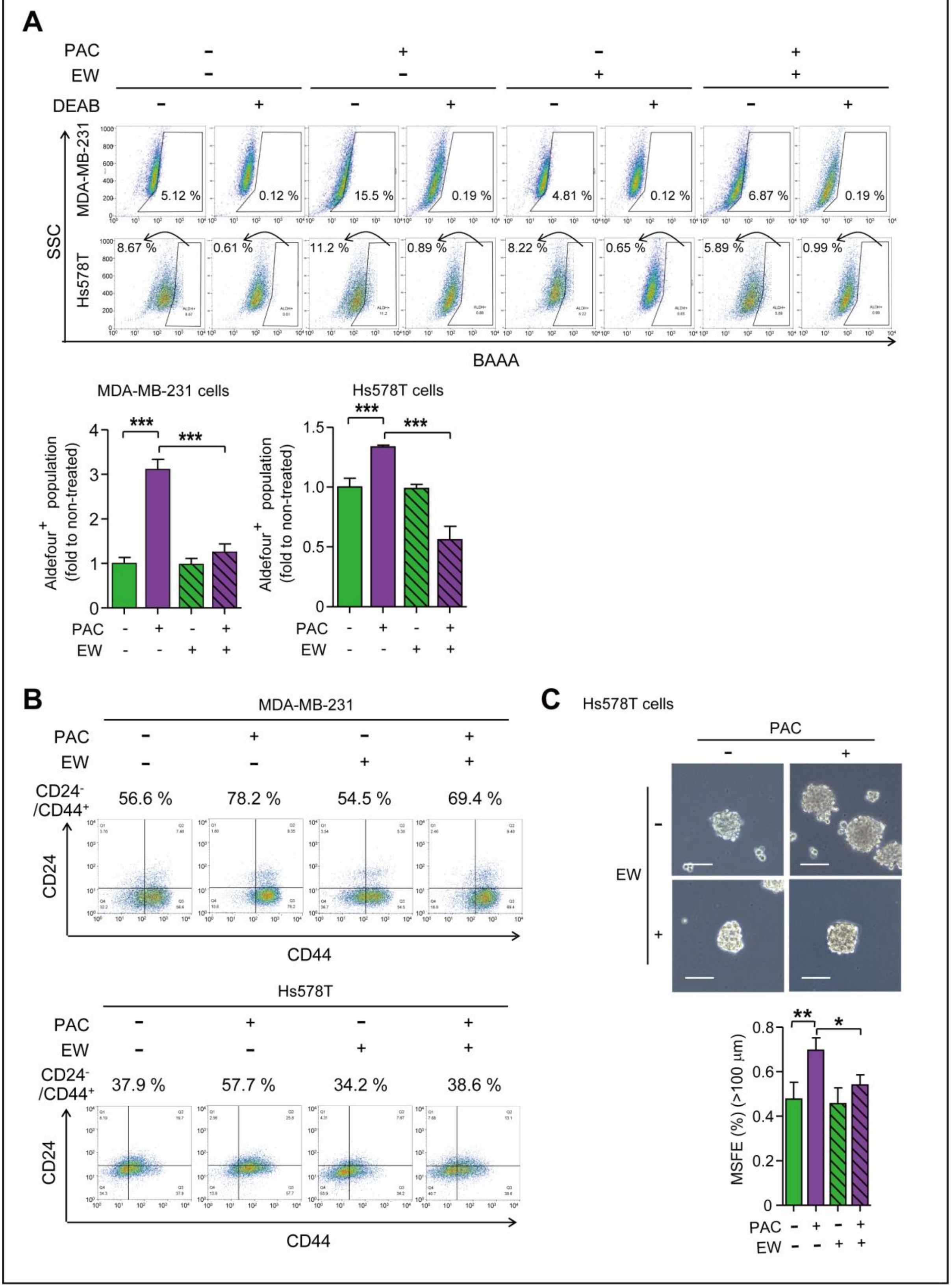

Supplementary Figure 6.

(A) The activity of ALDH was measured by Aldeflour^{TM} in MDA-MB-231 or Hs578T cells after 24-hour-treatmnet of paclitaxel (3 nM) with or without EW-7197 (100 nM). Upper panels show the representative 2D-plots of FACS analysis, and lower graphs show the ALDH-positive (ALDH^{+}) population as fold change compared to control (n= 3/group). (B) 2D-plots of FACS for analyzing CD24 and CD44 in MDA-MB-231 or Hs578T cells which were treated as the same as above (A). The percent of CD24-negative and CD44-positive (CD24^{-}/CD44^{+}) population is presented. (C) Sphere forming efficiency was calculated after the five-day-culturing Hs578T cells in the ultra-low attachment plates which were treated by paclitaxel (3 nM) with or without EW-7197 (100 nM) for 24 hours in attached-culture condition. The upper panels show the phase-contrast microscopy images of spheres (× 100, scale bar: 100 μm) and the graph shows the mammosphere-forming efficiency (MSFE) over 100 μm (n= 3/group). *, **, and *** indicate P<0.05, P<0.001, and P<0.005, respectively..

Supplementary Figure 7

A Taxane-treated breast cancer patients

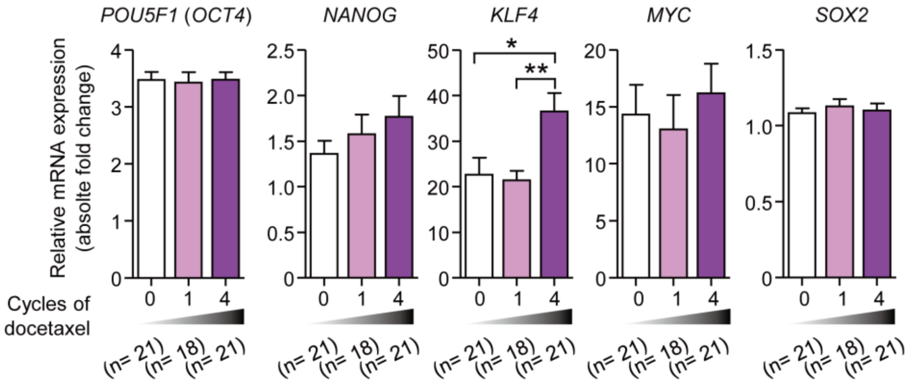

Supplementary Figure 7.

(A) mRNA expression levels of pluripotency regulators (*OCT4, NANOG, KLF4, MYC,* and SOX2) in breast cancer patients of Korde dataset from Oncomine (www. oncomine.com). (n=21, 18, 21, at 0-, 1-, 4-cycle of docetaxel, respectively) (** and *** indicate $P<0.01$ and $P<0.005$, respectively).

Supplementary Figure 8

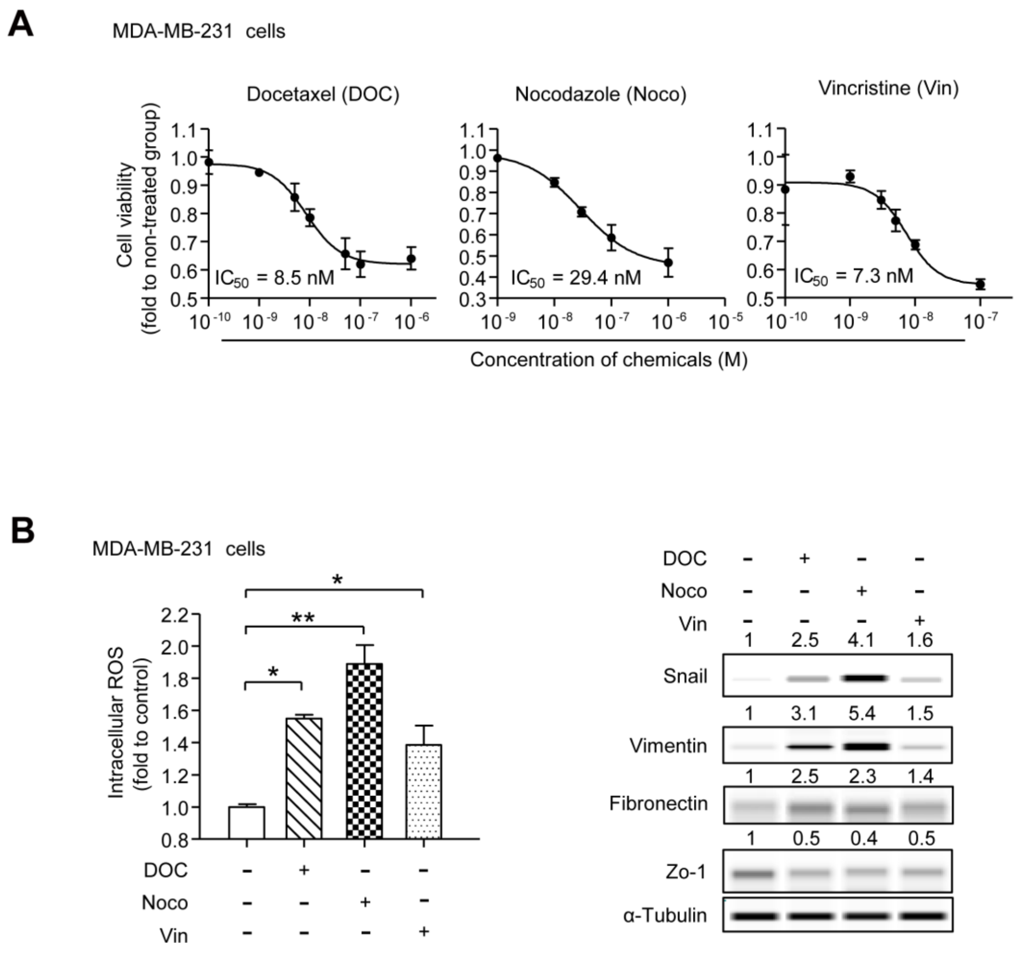

Supplementary Figure 8.

(A) IC_{50} values of docetaxel (DOC), nocodazole (Noc), and vincristine (Vin) were calculated by cell viability assays in MDA-MB-231 cells (described in Supplementary Material and Mthods). (B) MDA-MB-231 cells were treated with the IC_{50} concentrations of each chemical (8 nM for DOC, 30 nM for Noco, and 7 nM for Vin) and DCF-sensitive intracellular ROS were measured by a micro-plate reader (described in Supplementary Material and Methods). Also, MDA-MB-231 cells were treated with the IC_{50} concentrations of each chemicals for 24 hours in serum-reduced media (1% FBS). Whole protein lysates were subjected to protein analysis by Wes analysis. (*,** and *** indicate $P<0.05$, $P<0.01$ and $P<0.005$, respectively).
